# Dissection of the microRNA Network Regulating Hedgehog Signaling in *Drosophila*

**DOI:** 10.3389/fcell.2022.866491

**Published:** 2022-04-28

**Authors:** Tao He, Yu Fan, Yao Wang, Min Liu, Alan Jian Zhu

**Affiliations:** ^1^ Ministry of Education Key Laboratory of Cell Proliferation and Differentiation, School of Life Sciences, Peking University, Beijing, China; ^2^ Peking-Tsinghua Center for Life Sciences, Academy for Advanced Interdisciplinary Studies, Peking University, Beijing, China

**Keywords:** *Drosophila* wing, hedgehog signaling, *in vivo* miRNA sensor toolbox, *miR-10*, *miR-958*

## Abstract

The evolutionarily conserved Hedgehog (Hh) signaling plays a critical role in embryogenesis and adult tissue homeostasis. Aberrant Hh signaling often leads to various forms of developmental anomalies and cancer. Since altered microRNA (miRNA) expression is associated with developmental defects and tumorigenesis, it is not surprising that several miRNAs have been found to regulate Hh signaling. However, these miRNAs are mainly identified through small-scale *in vivo* screening or *in vitro* assays. As miRNAs preferentially reduce target gene expression *via* the 3′ untranslated region, we analyzed the effect of reduced expression of core components of the Hh signaling cascade on downstream signaling activity, and generated a transgenic *Drosophila* toolbox of *in vivo* miRNA sensors for core components of Hh signaling, including *hh*, *patched* (*ptc*), *smoothened* (*smo*), *costal 2* (*cos2*), *fused* (*fu*), *Suppressor of fused* (*Su(fu)*), and *cubitus interruptus* (*ci*). With these tools in hand, we performed a genome-wide *in vivo* miRNA overexpression screen in the developing *Drosophila* wing imaginal disc. Of the twelve miRNAs identified, seven were not previously reported in the *in vivo* Hh regulatory network. Moreover, these miRNAs may act as general regulators of Hh signaling, as their overexpression disrupts Hh signaling-mediated cyst stem cell maintenance during spermatogenesis. To identify direct targets of these newly discovered miRNAs, we used the miRNA sensor toolbox to show that *miR-10* and *miR-958* directly target *fu* and *smo*, respectively, while the other five miRNAs act through yet-to-be-identified targets other than the seven core components of Hh signaling described above. Importantly, through loss-of-function analysis, we found that endogenous *miR-10* and *miR-958* target *fu* and *smo*, respectively, whereas deletion of the other five miRNAs leads to altered expression of Hh signaling components, suggesting that these seven newly discovered miRNAs regulate Hh signaling *in vivo*. Given the powerful effects of these miRNAs on Hh signaling, we believe that identifying their *bona fide* targets of the other five miRNAs will help reveal important new players in the Hh regulatory network.

## Introduction

Hedgehog (Hh) signaling is a highly conserved pathway that controls multiple developmental processes, including pattern formation, proliferation and differentiation within diverse tissues. Dysfunction of Hh signaling can lead to birth defects, such as holoprosencephaly and cyclopia, and is associated with multiple cancer types, including medulloblastoma and basal cell carcinoma (Lee et al., 2016; Pak and Segal, 2016; Jeng et al., 2020; Liu M. et al., 2021; Jiang, 2021; Sigafoos et al., 2021). As a classic model system, the *Drosophila* wing plays a seminal and pivotal role in the delineation of the Hh signaling cascade. In the developing wing imaginal disc, the primordium of the adult wing, the ligand Hh acts as a morphogen. It is expressed in posterior compartment cells and secreted into the anterior compartment, where it binds to the cell surface receptor Patched (Ptc) and releases the signal transducer Smoothened (Smo) from inhibition. Activated Smo is transported from the endosome to the plasma membrane, during which it is sequentially phosphorylated by protein kinase A (PKA) and casein kinase 1 (CK1), and then recruits the motor protein Costal2 (Cos2), Fused (Fu) kinase, and Suppressor of fused (Su(fu)) to form an activation complex. As a result, a cytoplasmic signaling complex containing Cos2, Fu, Su(fu), and transcription factor Cubitus interruptus (Ci) is disassociated. Full-length Ci (Ci^FL^) becomes stable and enters the nucleus to activate the expression of downstream target genes, such as *decapentaplegic* (*dpp*), *ptc*, and *collier* (*col*) (Hartl and Scott, 2014; Liu M. et al., 2021; Jiang, 2021).

microRNAs (miRNAs) are endogenous noncoding RNAs of approximately 22 nucleotides in length. They mediate post-transcriptional gene repression through sequence-specific pairing between the miRNA seed sequence at positions 2–7 and the corresponding complementary sequence located primarily in the 3′ untranslated region (3′ UTR) of the mRNA target, thereby inhibiting mRNA translation, promoting mRNA decay, or both (Bushati and Cohen, 2007; Bartel, 2018). miRNAs have been found to regulate Hh signaling during development and tumorigenesis. Several miRNAs, including *miR-125b*, *miR-212*, *miR-324*, and *miR-326*, are misregulated in human medulloblastoma and pancreatic ductal adenocarcinoma, and may target key Hh signaling components to promote tumor cell proliferation and invasion (Ferretti et al., 2008; Ma et al., 2014). Among them, *miR-125b* and *miR-326* target *SMO*, and *miR-324* and *miR-212* target *GLI1* (ortholog of *ci*) and *PTCH1*, respectively (Ferretti et al., 2008; Ma et al., 2014). Furthermore, *miR-30c* was reported to suppress mouse P19 cell differentiation by targeting *Gli2* (Liu et al., 2016). In zebrafish, *miR-214* targets *Su(fu)* and is required for precise specification of slow-muscle cell types (Flynt et al., 2007). In *Drosophila*, *smo*, *cos2*, and *fu* are targets of *miR-12* and *miR-283* (Friggi-Grelin et al., 2008). *miR-7* and *miR-14* negatively regulate Hh signaling by targeting *interference hedgehog* (*ihog*), a co-receptor of Hh ligand, and *hh*, respectively (Da Ros et al., 2013; Kim et al., 2014). In addition, two screens have been performed to identify miRNAs that regulate Hh signaling. The first screen was a small scale miRNA overexpression screen examining the role of 40 miRNAs in *Drosophila* wings and found that *miR-5*, *miR-932*, and *miR-960* modulate Hh signaling by directly targeting *smo*, *brother of ihog* (*boi*), and *smo*, *cos2* and *fu*, respectively (Wu et al., 2012; Gao et al., 2013a; Gao et al., 2013b). The second screen analyzed 132 miRNAs in cultured *Drosophila* S2R^+^ cells. Using *in vitro* miRNA sensors for core Hh pathway components as readouts, 43 miRNAs were identified as potential regulators of Hh signaling (Kim et al., 2014). However, these early screens were either poorly covered or not fully validated *in vivo*. For example, *miR-5* and *miR-960*, which are known to regulate Hh signaling in wing discs, were not identified in the *in vitro* screen (Kim et al., 2014). *miR-7* and *miR-932* were reported in the *in vivo* screen to target *ihog* and *boi*, respectively (Gao et al., 2013b; Da Ros et al., 2013), but *in vitro* screening only found them to target *cos2* and *hh*, respectively (Kim et al., 2014). Furthermore, *miR-12* and *miR-283* target multiple Hh pathway components in the developing wing, but were shown to regulate only a single target in the *in vitro* screen (Friggi-Grelin et al., 2008; Kim et al., 2014). These disagreements suggest that *in vitro* screening may not be sufficient to identify miRNAs that regulate Hh signaling *in vivo*. Therefore, a genome-wide *in vivo* screen is required to systematically assess the *in vivo* role of miRNAs in Hh signaling.

In this study, we performed a genome-wide miRNA overexpression screen in *Drosophila* wing discs to dissect the miRNA network regulating Hh signaling. By examining the effects of overexpressed miRNAs on Hh signaling activation, such as Ci^FL^ stabilization and *col* activation, five out of the seven miRNAs known to regulate Hh signaling *in vivo* were identified. Moreover, our screen added seven additional miRNAs, namely *miR-10*, *miR-133*, *miR-190*, *miR-375*, *miR-927*, *miR-958*, and *miR-964*, to the miRNA network regulating Hh signaling. These newly discovered miRNAs all regulate Hh signaling in a cell-autonomous manner, as revealed by FLIPout clonal analysis. Furthermore, these miRNAs can regulate Hh signaling in the *Drosophila* testis, suggesting that they may act as general regulators of Hh signaling. Using the *in vivo* miRNA sensor transgenic fly toolbox, we provided direct evidence that *miR-10* and *miR-958* target *fu* and *smo*, respectively. Consistently, deletion of *miR-10* and *miR-958* significantly increased the expression of *fu* and *smo*, respectively. We have not yet identified the direct targets of the remaining five miRNAs in the Hh pathway regulatory network. However, aberrant expression of Hh pathway components was observed in loss-of-function alleles of these five miRNAs, suggesting that these newly identified miRNAs are physiologically required to maintain Hh signaling homeostasis. Therefore, finding their targets may lead to the discovery of new players in the regulatory network of Hh signaling associated with development and Hh-related diseases.

## Results

### The Developing *Drosophila* Wing as an Ideal Model System for Dissecting the miRNA Network That Regulates Hh Signaling

**FIGURE 1:**
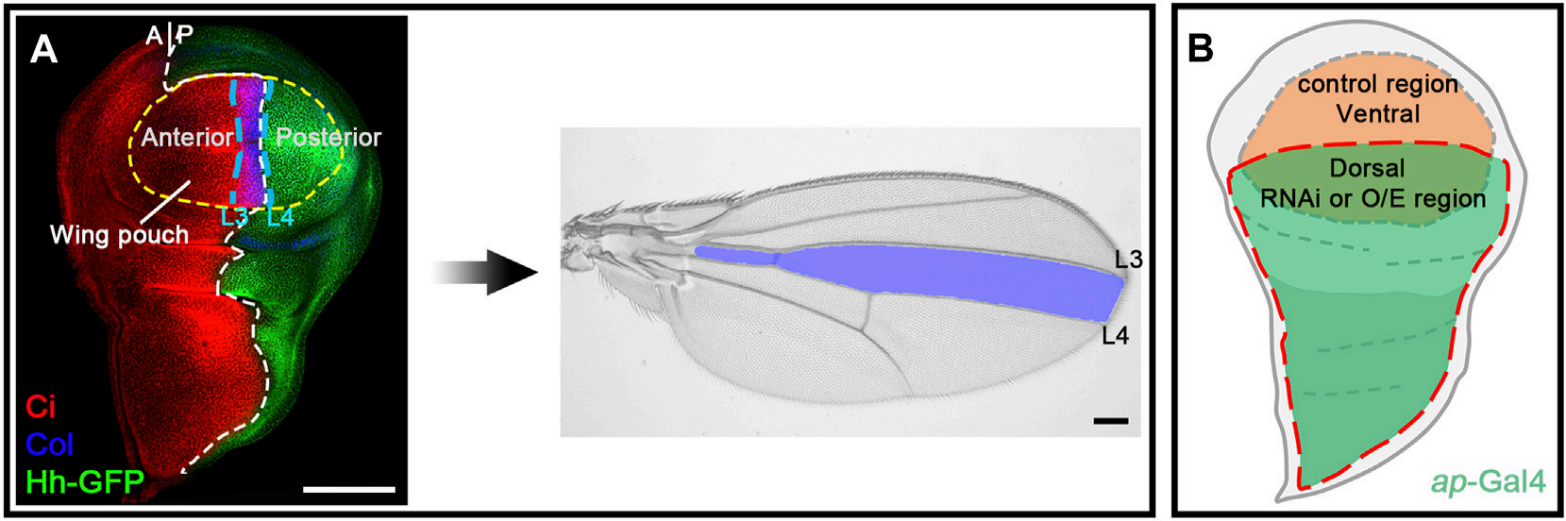
The developing *Drosophila* wing is an ideal model system for studying Hh signaling. **(A)** Shown are a wild-type third instar larval wing imaginal disc and an adult wing blade. Expression of Hh-GFP (green), Col (blue), and Ci (red) is shown. Hh protein is produced in the posterior compartment and diffuses into the anterior compartment to stabilize Ci^FL^ protein and activate *col* expression. The wing pouch (outlined by a yellow dashed circle) eventually metamorphose into the adult wing blade. The area between the longitudinal L3 and L4 veins of the adult wing is predetermined in the area between the two blue dashed lines in the larval wing disc, where Col is expressed. **(B)** A diagram showing *ap*-Gal4 expression area. Cells in the ventral compartment were used as an internal control. Scale bar, 100 μm.

The adult *Drosophila* wing consists of five longitudinal veins that intersect with two transverse crossveins, extending distally to the wing margin. This stereotypical adult wing morphology is pre-patterned during larval development in a primordial tissue called the wing imaginal disc (Blair, 2007; Hartl and Scott, 2014). Hh glycoprotein, secreted from the posterior half of the wing disc, acts as a morphogen to control patterning and cell proliferation in the anterior half of the wing disc. Anterior cells abutting the anterior-posterior (A-P) boundary are sensitive to Hh signaling activity. Hh morphogen stabilizes Ci^FL^ protein to activate transcription of *col*/*kn*, which encodes a transcription factor required for patterning a region in the wing pouch corresponding to the area between longitudinal L3 and L4 veins in the adult wing blade (Blair, 2007; Hartl and Scott, 2014; Figure 1A). As the loss of *ci* or *col* expression in the wing disc results in a reduction in the space between the L3 and L4 veins (Vervoort et al., 1999), stabilization of Ci^FL^ and activation of *col* transcription are ideal indicators of Hh signaling activity, which can be easily determined by immunostaining with specific antibodies or through enhancer trap transgenic reporters. Monoclonal antibody 2A1 is commonly used to monitor Ci^FL^ stability (Motzny and Holmgren, 1995). Notably, in anterior cells immediately adjacent to the A-P boundary, stabilized Ci^FL^ is converted to the labile activated form of Ci (Ci^A^). Consequently, the protein levels of 2A1-labeled Ci^FL^ are relatively low in these cells (Motzny and Holmgren, 1995; Aza-Blanc et al., 1997; Ohlmeyer and Kalderon, 1998). *col* transcription can be monitored by a polyclonal antibody specific for Col, or visualized in the activity of the *kn*-lacZ transgenic reporter, whose expression is controlled by the *kn* ^*Mel*^ *701-1991* regulatory element (Hersh and Carroll, 2005).

**FIGURE 2:**
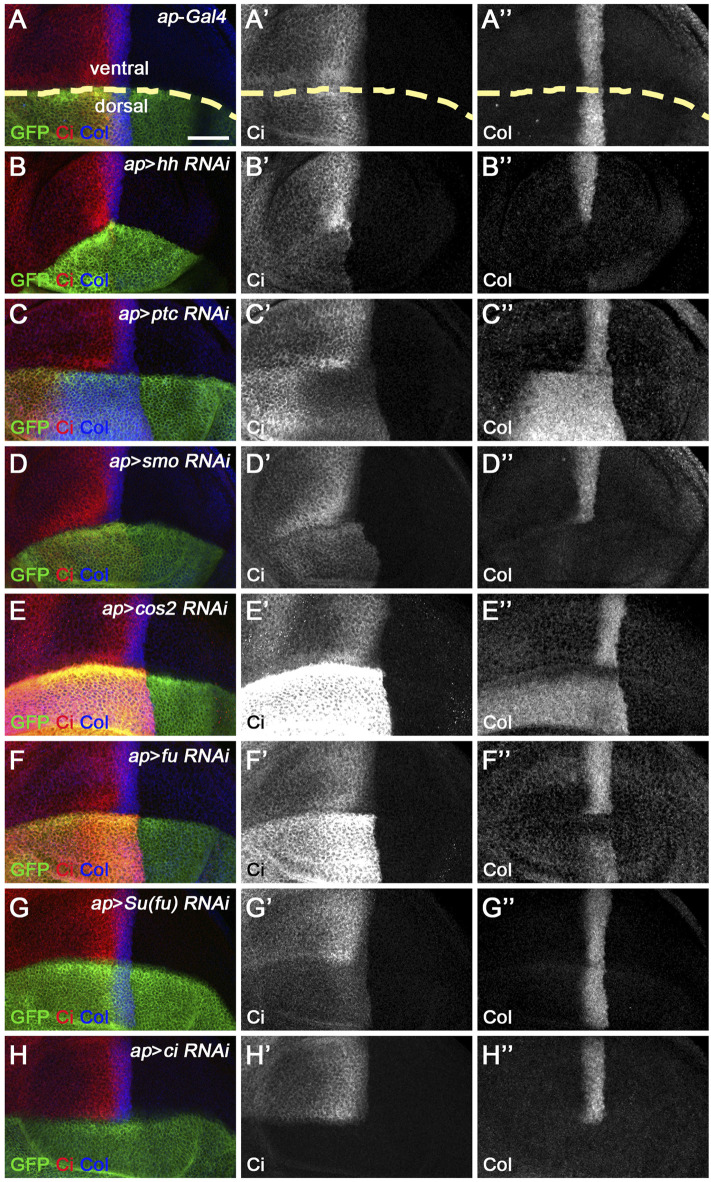
Knockdown of core Hh pathway components alters Ci^FL^ levels and Col expression. Ci^FL^ levels and Col expression were visualized by immunostaining in third instar larval wing discs of the indicated genotypes. GFP marks dsRNA-expressing cells in the dorsal compartment. **(A–B’’,D–D’’,H–H’’)** *ap*-Gal4-driven RNAi of the positive regulators of Hh signaling (i.e. *hh*, *smo*, and *ci*) resulted in significantly reduced levels of Ci^FL^ **(B’,D’)** and loss of Col expression **(B”,D”,H”)** in dorsal compartment cells. **(C–C’’,E–E’’)** Conversely, knockdown of *ptc* caused expansion of the labile Ci^A^ region **(C’)** and expansion and increase in Col expression **(C”)**. Significant increases in Ci^FL^ levels **(E’)** and expanded Col expression **(E”)** were observed when *cos2* was knocked down. **(F–G’’)** Decreased expression of *fu* resulted in elevated levels of Ci^FL^ **(F’)** and downregulation of Col **(F”)**, whereas knockdown of *Su(fu)* led to significantly decreased levels of Ci^FL^ **(G’)** but had no effects on Col expression **(G”)**. The dorsal-ventral boundary of the wing disc is marked with a yellow dashed line. Scale bar, 50 μm.

Since miRNAs typically repress the expression of target genes, overexpressed miRNAs that directly target the Hh pathway may reduce the expression of genes encoding core Hh signaling components that contain miRNA-binding sites. As Ci^FL^ protein stabilization and *col* transcription are convenient and reliable readouts for Hh signaling activity, we reduced the expression of individual canonical Hh pathway components to analyze their effects on Ci^FL^ and Col for a comprehensive understanding of miRNA regulation in Hh signaling. Among the core components examined, Hh, Smo, and Ci are positive regulators, and Hh signaling activity is impaired when the genes encoding these positive regulators are mutated (Alexandre et al., 1996; van den Heuvel and Ingham, 1996; Strigini and Cohen, 1997; Vervoort et al., 1999). Consistently, when these genes were individually knocked down by *apterous* (*ap*)-Gal4-driven RNAi in dorsal compartment cells (Figure 1A and Figure 2A), we observed a significant reduction in the protein levels of Ci^FL^ and abrogation of Col expression (Figures 2B–B’’,D–D’’,H–H’’). Ptc and Cos2 negatively regulate Hh signaling by inhibiting the activation of Smo and promoting the degradation of Ci, respectively (Chen and Struhl, 1996; Sisson et al., 1997; Alcedo et al., 2000; Denef et al., 2000; Ingham et al., 2000; Zhu et al., 2003). Consistent with these studies, knockdown of *ptc* resulted in expansion of the labile Ci^A^ region as well as expansion and elevation of Col expression in dorsal compartment cells (Figures 2C–C’’). Likewise, reduction of *cos2* expression by RNAi caused a marked increase in Ci^FL^ protein levels and an expansion of the Col-expression region (Figures 2E–E’’). The roles of Fu and Su(fu) in Hh signaling are more complex. On the one hand, Fu is required to transduce high levels of Hh signaling activity by converting Ci^FL^ to Ci^A^ (Ohlmeyer and Kalderon, 1998; Han et al., 2019). On the other hand, it also cooperates with Cos2 to promote the degradation of Ci^FL^ (Wang et al., 2000; Zhang et al., 2005). In the wing discs of *fu* mutant flies, Ci^FL^ levels are elevated but, unexpectedly, Col expression is attenuated and labile Ci^A^ disappears (Alves et al., 1998). We observed similar results in dorsal compartment cells, where *fu* was knocked down by *ap*-Gal4-driven RNAi (Figures 2F–F’’). Su(fu) antagonizes the function of Fu, and reduced levels of Ci^FL^ are observed in *Su(fu)* mutants (Préat, 1992; Alves et al., 1998; Ohlmeyer and Kalderon, 1998). However, loss of *Su(fu)* does not result in ectopic activation of Hh signaling nor any apparent defects in adult wings (Préat et al., 1993). Consistent with previous studies, knockdown of *Su(fu)* in the dorsal compartment had no effects on Col expression, although decreased levels of Ci^FL^ protein were observed (Figures 2G–G’’).

### The Generation of an *in vivo* miRNA Sensor Toolbox for Core Hh Signaling Components

Since miRNAs primarily act by reducing target gene expression, a direct comparison of the impact of overproduced miRNAs with that of reduced expression of individual components of the Hh pathway will help identify specific Hh signaling components as potential miRNA targets. In addition, *in vivo* miRNA sensors containing 3′ UTRs of known Hh signaling players will provide clear evidence that candidate miRNAs function by directly targeting the corresponding core Hh pathway components (Brennecke et al., 2003). Previously, *in vivo* miRNA sensors for *smo*, *cos2*, and *fu* have been generated and used to identify these genes individually or in combination as direct targets of *miR-5*, *miR-12*, *miR-283*, or *miR-960* (Friggi-Grelin et al., 2008; Wu et al., 2012; Gao et al., 2013a).

To extend the coverage of *in vivo* miRNA sensors to all known core components of the Hh pathway, we added four additional miRNA sensors for *hh*, *ptc*, *Su(fu)*, and *ci* to assemble an *in vivo* miRNA sensor toolbox for Hh signaling. In these miRNA sensors, the expression of *gfp* reporter is controlled by the *αTub84B* promoter and the 3′ UTR derived from *hh*, *ptc*, *smo*, *cos2*, *fu*, *Su(fu)*, or *ci* (Supplementary Figure S1A). Transgenic flies were generated through φC31 integrase-mediated site-directed integration. As expected, ubiquitous GFP expression was observed for all seven miRNA sensors in the wing disc (Supplementary Figures S1B–H’’). To demonstrate the effectiveness of these miRNA sensors, we used *dpp*-Gal4 to overexpress *miR-14*, *miR-960*, and *miR-12*, which are known to target *hh*, *smo*, and *cos2* and *fu*, respectively, in anterior cells abutting the A-P boundary (Friggi-Grelin et al., 2008; Gao et al., 2013a; Kim et al., 2014). Consistent with previous reports, overexpression of *miR-14* caused a mild but consistent downregulation of the miRNA sensor activity for *hh* (*gfp*: *3′UTR* ^*hh*^) (Supplementary Figures S2A–A’’). Increased *miR-960* expression by *ptc*-Gal4 resulted in a significant decrease in *gfp*: *3′UTR* ^*smo*^ activity and Smo protein levels (Supplementary Figures S2B–B’’). *miR-12* is known to directly target *cos2* and *fu*. Consistently, increased *miR-12* expression led to significantly decreased expression of *gfp*: *3*′*UTR* ^*cos2*^ and *gfp*: *3*′*UTR* ^*fu*^ (Supplementary Figures S2C–D’’). Although the remaining three miRNA sensors for *ptc*, *Su(fu)*, and *ci* were not examined because no miRNAs targeting these genes were reported, the above results suggest that the assembled *in vivo* miRNA sensor toolbox can be used to determine whether candidate miRNAs directly target core Hh pathway genes.

### A Targeted Genome-Wide *in vivo* miRNA Overexpression Screen to Identify Novel miRNAs Regulating Hh Signaling

Using these two tools, we performed a genome-wide *in vivo* miRNA overexpression screen to systematically assess the regulation of Hh signaling by miRNAs. The *Drosophila melanogaster* genome contains 258 miRNA precursors, resulting in 469 mature miRNA sequences, most of which were determined by miRNA sequencing and bioinformatics prediction (Griffiths-Jones et al., 2008; Kozomara and Griffiths-Jones, 2011). In our screen, we analyzed more than 97% of miRNA precursors (149 out of 153) whose expression was confirmed by miRNA sequencing *in vivo* (Kozomara and Griffiths-Jones, 2014; Larkin et al., 2021). Of note, we did not include *miR-283*, *miR-9369*, *miR-9388*, and *miR-10404* in the screen because no transgenic flies were available. Furthermore, our screen included 30 additional miRNAs whose expression *in vivo* had not been validated. Together, we screened 190 transgenic overexpression fly lines, covering a total of 179 miRNA precursors (hereafter referred to as miRNAs), to dissect their roles in Hh signaling (Supplementary Table S1). To unbiasedly assess the impact of individual miRNAs in Hh signaling, miRNA overexpression was confined to the dorsal half of the wing disc using the *ap*-Gal4 driver, whereas unaffected wild-type cells in the ventral half of the wing disc served as a perfect internal control for miRNA overexpression (Figure 1B).

**FIGURE 3:**
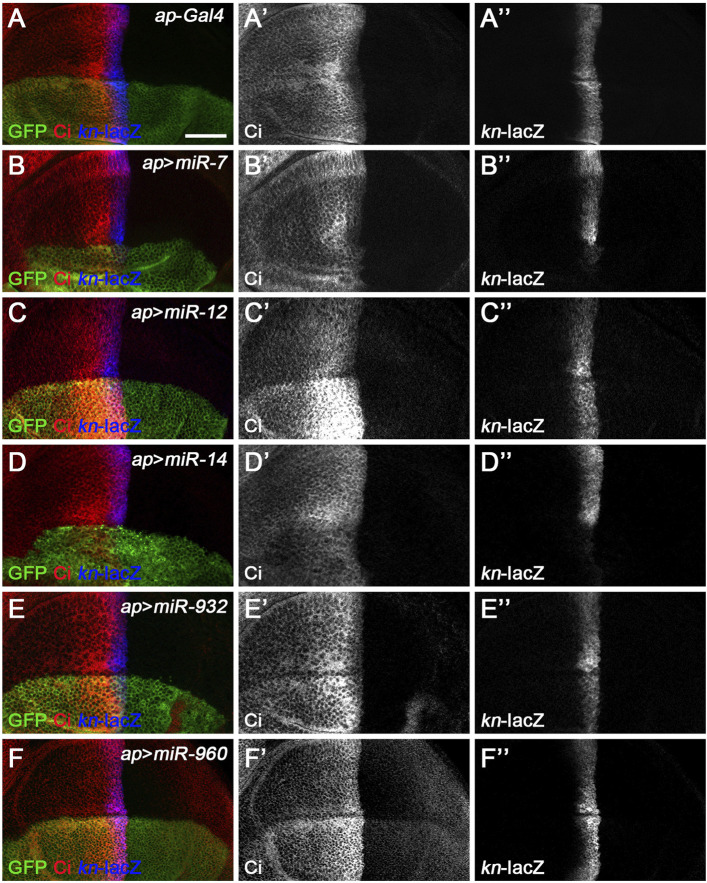
Overexpression of previously known Hh signaling-regulating miRNAs alters Ci^FL^ levels and *kn*-lacZ reporter activity. Ci^FL^ levels and *kn*-lacZ activity were monitored by immunostaining in third instar larval wing discs of the indicated genotypes. **(A–B’’,D–D’’)** *ap*-Gal4-driven overexpression of *miR-7* or *miR-14* resulted in decreased levels of Ci^FL^ **(B’,D’)** and loss of *kn*-lacZ expression **(B”,D”)** in the dorsal compartment. **(C–C’’,F–F’’)** Increased expression of *miR-12* or *miR-960* led to markedly elevated levels of Ci^FL^ **(C’,F’)**, but little change in *kn*-lacZ activity **(C”,F”)**. **(E–E’’)** A mild increase in Ci^FL^ levels **(E’)** and a weak downregulation of *kn*-lacZ activity **(E”)** were observed in GFP-positive cells overexpressing *miR-932*. Scale bar, 50 μm.

In our screen, five of the seven miRNAs previously reported to regulate Hh signaling *in vivo* were shown to affect the expression of Ci and/or *kn*-lacZ (Figure 3). Among these miRNAs, *miR-7*, *miR-14*, and *miR-932* negatively regulate Hh signaling by targeting *ihog*, *hh*, and *boi*, respectively (Table 1). Consistent with previous reports (Wu et al., 2012; Da Ros et al., 2013; Gao et al., 2013b; Kim et al., 2014), overexpression of *miR-7* or *miR-14* resulted in decreased levels of Ci^FL^ and downstream target *col* expression, compared with wild-type cells in the ventral half of the disc (Figures 3B–B’’,D–D’’). Likewise, increased *miR-932* expression led to a modest increase in Ci^FL^ protein levels and a narrowing of the Col-expression domain (Figures 3E–E’’). As described previously, both *miR-12* and *miR-960* target multiple genes encoding components of the Hh signaling pathway, namely *smo*, *cos2*, and *fu* (Table 1). Overexpression of *miR-12* induced elevated levels of Ci^FL^, but not enough to alter the expression of the *dpp*-lacZ reporter (Friggi-Grelin et al., 2008). Consistently, we observed increased levels of Ci^FL^ and little change in *kn*-lacZ expression when *miR-12* was overexpressed by the *ap*-Gal4 in the dorsal compartment (Figure 3C–C’’). Although the effect of overexpressed *miR-960* on Ci protein stability and *col* expression was not examined in the previous study (Gao et al., 2013a), given that it shares the same targets as *miR-12*, we speculated that *miR-960* may regulate Hh signaling in a similar manner to *miR-12.* Indeed, overexpression of *miR-960* resulted in an obvious increase in Ci^FL^ levels, while *kn*-lacZ expression remained unchanged (Figures 3F–F”). These results demonstrate that our screen using the *Drosophila* wing disc as an *in vivo* model platform is robust and sensitive to identify Hh signaling-regulating miRNAs.

**TABLE 1:** miRNAs previously reported to regulate Hh signaling *in vivo.*

miRNAs	Targets	Functions	References
*miR-5*	*smo*	*miR-5* overexpression reduces the intervein region between L3 and L4 in the adult wing	Wu et al. (2012)
*miR-7*	*ihog*	*miR-7* overexpression facilitates notch-induced tumorigenesis in the eye	Da Ros et al. (2013)
*miR-12*	*smo*, *cos2*, *fu*	Induces anterior wing outgrowth when co-expressed with *miR-283*	Friggi-Grelin et al. (2008)
*miR-14*	*hh*	Modulates wing size and ensures the correct number of tracheal terminal cells	Kim et al. (2014)
*miR-283*	*smo*, *cos2*, *fu*	Induces anterior wing outgrowth when co-expressed with *miR-12*	Friggi-Grelin et al. (2008)
*miR-932*	*boi*	*miR-932* overexpression reduces the intervein region between L3 and L4 in the adult wing	Gao et al. (2013a)
*miR-960*	*smo*, *cos2*, *fu*	Reduces the intervein region between L3 and L4 in the adult wing when co-expressed with *miR-959*, *miR-961*, and *miR-962*	Gao et al. (2013b)

In addition to the previously identified miRNAs, we discovered seven additional miRNAs, namely *miR-10*, *miR-133*, *miR-190*, *miR-375*, *miR-927*, *miR-958*, and *miR-964*, as novel regulators of Hh signaling. All of these newly discovered miRNAs have detectable expression in larval imaginal discs, with *miR-964* being the most highly expressed miRNA, 8-fold higher than the other six miRNAs (Ruby et al., 2007). Furthermore, *miR-964* was found to be specifically enriched in imaginal tissues (Ruby et al., 2007; Supplementary Table S2). When comparing our screen with previously reported miRNA screens, we found that *miR-5*, reported to target *smo* (Wu et al., 2012), was not identified in our screen, nor was it found in a miRNA screen conducted *in vitro* in S2R^+^ cells (Kim et al., 2014). Overexpression of *miR-5* had no obvious effects on the expression of Smo, Ci, or *kn*-lacZ (Supplementary Figure S3). This observation was further validated in S2R^+^ cells, as increased *miR-5* expression did not have any apparent effect on the activity of the miRNA sensor for *smo* (Kim et al., 2014). This inconsistency may be due to the different transgenic flies used for miRNA overexpression (Wu et al., 2012). We further noted that among the seven newly identified miRNAs, *miR-10*, *miR-133*, *miR-927*, and *miR-964* appeared in our screen as well as in the *in vitro* screen with S2R^+^ cells, while the other three miRNAs, *miR-190*, *miR-375*, and *miR-958*, were only identified in our screen, suggesting that the *in vitro* screen in hemocyte-like S2R^+^ cells (Schneider, 1972) may not be sufficient to identify miRNAs that regulate Hh signaling in wing discs.

### *miR-10* Negatively Regulates Hh Signaling by Targeting *fu*

**FIGURE 4:**
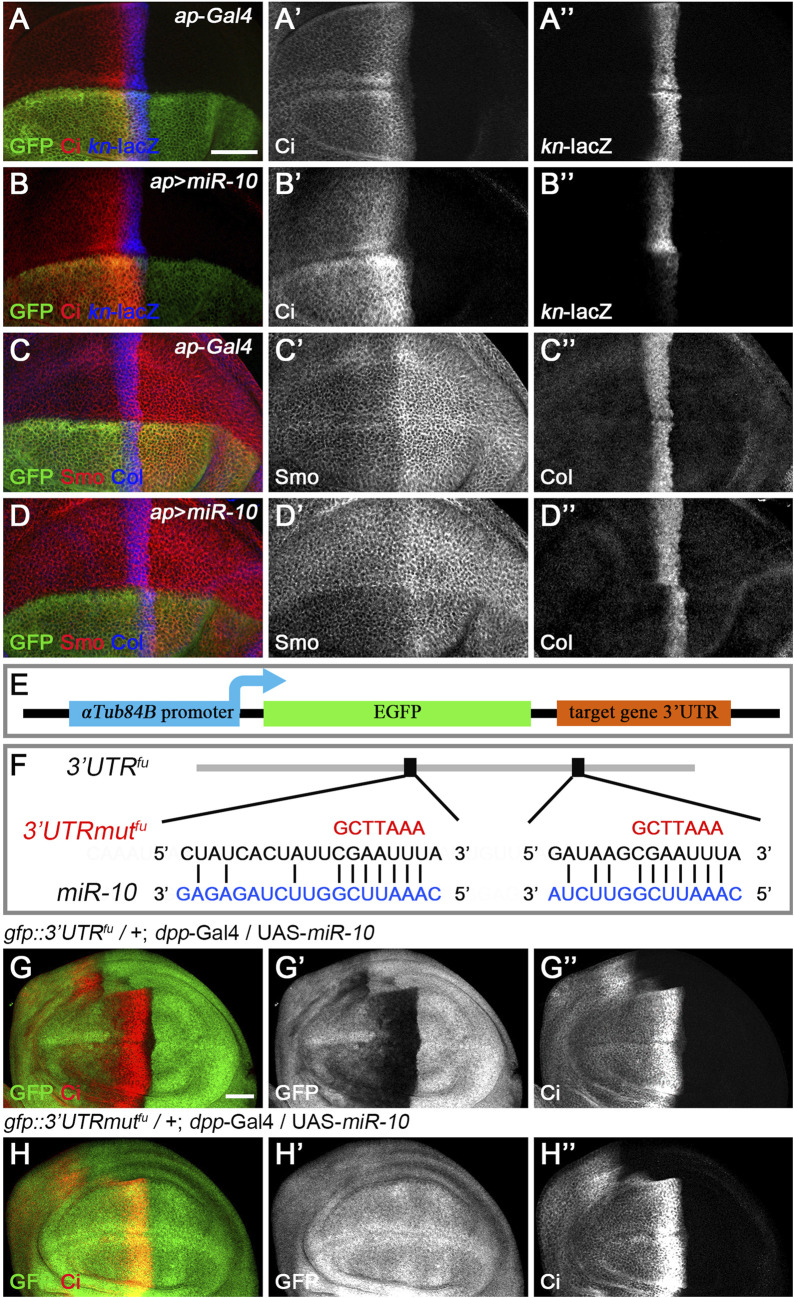
*miR-10* negatively regulates Hh signaling by targeting *fu*. **(A–B”)** Overexpression of *miR-10* by *ap*-Gal4 in the dorsal compartment of the wing disc resulted in an obvious increase in Ci^FL^ levels **(B’)** and a significant decrease in *kn*-lacZ activity **(B”)**. Smo protein levels were significantly reduced in both anterior and posterior compartment cells **(D’)**, and the expression region of Col was narrowed **(D”). (E)** Schematic showing the composition of a miRNA sensor containing the *αTub84B* promoter, the *egfp* coding sequence, and the 3′ UTR of individual core Hh pathway genes. **(F)** Two predicted *miR-10* binding sites are present in the 3′ UTR of *fu*. Mutated binding sites are shown in red. **(G–H”)** GFP expression of *gfp*: *3′UTR* ^*fu*^ and *gfp*: *3′UTRmut* ^*fu*^ sensor lines in *dpp*-Gal4-driven *miR-10* overexpression wing discs are shown. Increased *miR-10* expression resulted in significantly reduced GFP expression **(G′)** and elevated Ci^FL^ levels **(G”)** along the A-P boundary in *gfp*: *3′UTR* ^*fu*^ sensor wing discs. In contrast, overexpressed *miR-10* did not affect GFP expression in *gfp*: *3′UTRmut* ^*fu*^ sensor wing discs **(H’)**, although Ci^FL^ was upregulated **(H”)**. Scale bar, 50 μm.

Based on their effects on Ci^FL^ stability and *col* target gene transcription in wing discs, we classified the newly discovered miRNAs into three categories. *miR-10* belongs to the first category. Overexpression of *miR-10* resulted in elevated levels of Ci^FL^ protein at the expense of labile Ci^A^ (Figure 4B’), an effect likely due to decreased Hh signaling activity at the highest level. In addition, decreased *kn*-lacZ reporter activity and Col protein levels by anti-Col antibody staining were detected (Figures 4B’’,D’’), confirming the effect of reducing the highest level of Hh signaling. These results were also observed in wing disc cells, where *fu* function was impaired in loss-of-function mutants (Alves et al., 1998) or by RNAi (Figures 2F–F”). Therefore, *miR-10* could act through *fu*. We tested this hypothesis using the *in vivo gfp*: *3*′*UTR* ^*fu*^ miRNA sensor transgenic flies. Overexpression of *miR-10* by *dpp*-Gal4 along the A-P boundary resulted in a significant decrease in *gfp*: *3*′*UTR* ^*fu*^ sensor activity (Figures 4G–G”), indicating that *fu* is one of the direct targets of *miR-10*. Bioinformatics analysis (http://www.targetscan.org/) further supported this conclusion by identifying two putative *miR-10* binding sites in the 3′ UTR of *fu* (Figure 4F). To verify that *miR-10* regulates Hh signaling through these two miRNA binding sites, we generated *gfp*: *3*′*UTRmut* ^*fu*^ in which the *miR-10* binding sites in the 3’ UTR of *fu* were mutated (Figure 4F). We found that increased *miR-10* expression was no longer able to reduce *gfp*: *3*′*UTRmut* ^*fu*^ sensor activity (Figures 4H–H”). As altered Smo phosphorylation and activity may lead to changes in the highest levels of Hh signaling activity, we tested whether *miR-10* regulated Hh signaling by targeting other core Hh pathway genes. *miR-10* was overexpressed along the A-P boundary in the wing disc of miRNA sensor flies for *hh*, *ptc*, *smo*, *cos2*, *Su(fu)*, and *ci*, respectively. We found that *miR-10* failed to reduce the activity of any miRNA sensor other than *fu* in our assembled miRNA sensor toolbox (Supplementary Figure S4). Taken together, *miR-10* negatively regulates Hh signaling by specifically targeting *fu*.

### *miR-958* Acts as a Negative Regulator of Hh Signaling by Targeting *smo*

**FIGURE 5:**
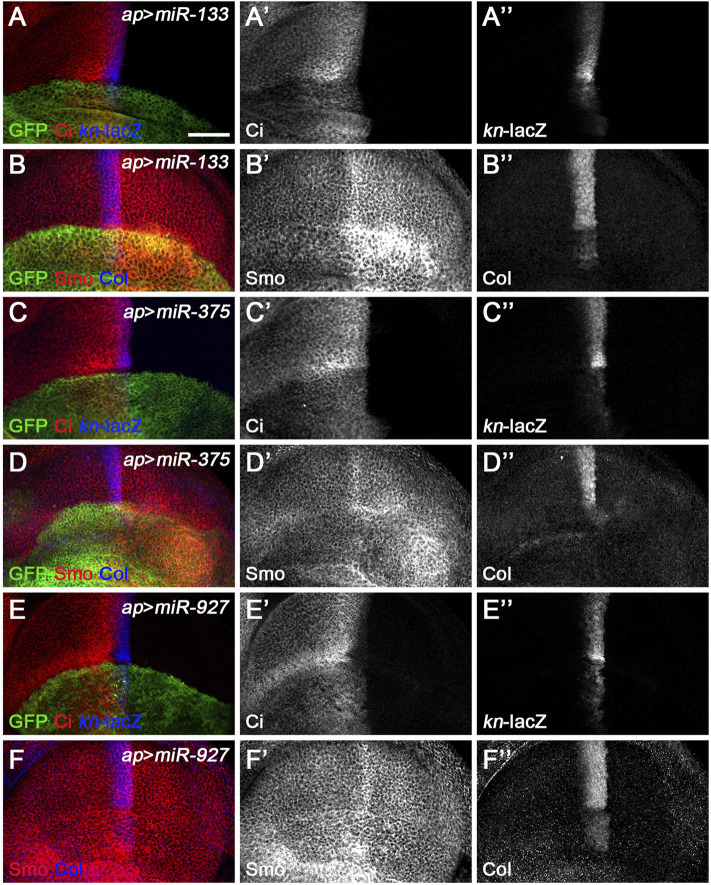
*miR-133*, *miR-375*, and *miR-927* are negative regulators of Hh signaling. Smo, Ci^FL^, and Col levels and *kn*-lacZ activity were visualized by immunostaining in wing discs overexpressing *miR-133* **(A–B”)**, *miR-375* **(C–D”)**, or *miR-927* **(E–F”)**. GFP marks the expression region of *ap*-Gal4. Although overexpression of *miR-133*, *miR-375*, and *miR-927* all resulted in significant decrease in Ci^FL^ levels **(A’,C’,E’)**, *kn*-lacZ activity **(A”,C”,E”)**, and Col expression **(B”,D”,F”)**, different effects on Smo expression were observed. Increased *miR-133* expression led to a marked increase in Smo protein levels in the posterior compartment, whereas the increase in the anterior compartment was very mild **(B’)**. It is difficult to determine the effect of *miR-375* overexpression on Smo expression because the formation of the dorsal compartment of the wing disc was disrupted **(D’)**. Overexpression of *miR-927* resulted in mildly elevated Smo levels, especially in the anterior compartment **(F’)**. Scale bar, 50 μm.

**FIGURE 6:**
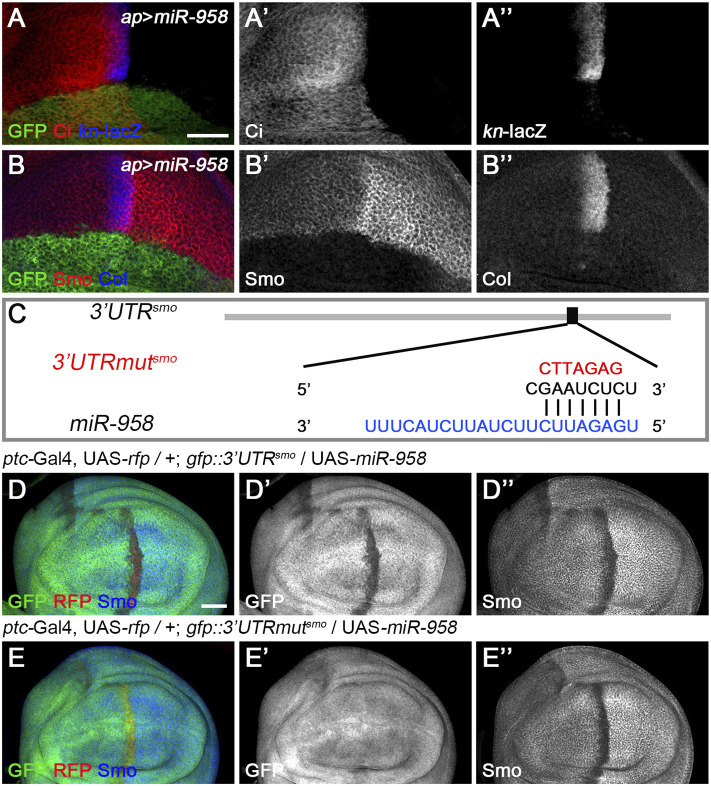
*miR-958* targets *smo* to regulate Hh signaling **(A–B”)** *ap*-Gal4-driven overexpression of *miR-958* in the dorsal compartment resulted in a marked reduction in Ci^FL^ levels **(A’)**, abrogation of Smo expression **(B’)** and complete loss of *kn*-lacZ activity **(A”)** and Col expression **(B”)**. **(C)** The predicted *miR-958* binding site is present in the 3′ UTR of *smo*, and the mutated binding site is shown in red **(D–E”)** GFP expression in wing discs of *gfp*: *3′UTR* ^*smo*^ and *gfp*: *3′UTRmut* ^*smo*^ sensor lines is shown when *miR-958* was overexpressed. *ptc*-Gal4-driven overexpression of *miR-958* resulted in obviously reduced GFP **(D’)** and Smo expression **(D”)** along the A-P boundary in the *gfp*:*3’UTR* ^*fu*^ sensor wing disc. In contrast, increased *miR-958* expression had no effects on GFP expression in the *gfp*: *3′UTRmut* ^*fu*^ sensor wing disc **(E’)**, although Smo protein levels were significantly reduced **(E”)**. Scale bar, 50 μm.

*miR-133*, *miR-375*, *miR-927*, and *miR-958* belong to the second class of Hh signaling-regulating miRNAs, as overexpression of these miRNAs by *ap*-Gal4 in dorsal compartment cells all resulted in significantly reduced levels of Ci^FL^, and *kn*-lacZ reporter activity and Col protein levels were completely lost (Figures 5A–A”,B”,C–C”,D”,E–E”,F”, Figure 6A–A’’,B’’). The above strongly reduced Hh signaling may be due to reduced expression of key positive regulators of Hh signaling, such as *hh*, *smo*, and *ci*. Since we did not have a working Hh antibody, we only examined the effect of overexpressing miRNAs on Smo and Ci levels. Overexpression of *miR-958* in the dorsal compartment of the wing disc completely abolished Smo expression (Figure 6B’), whereas *miR-133* had the opposite effect, resulting in a significant upregulation of Smo protein levels (Figure 5B’). In contrast to the homogeneous effects of *miR-958* and *miR-133* on Smo protein present in both anterior and posterior compartments*,* overexpressed *miR-927* only increased Smo protein levels in anterior compartment cells; the posteriorly localized Smo remained unchanged (Figure 5F’). The effects of *miR-375* overexpression were more complex. Since increased *miR-375* activity perturbed wing disc formation, it was difficult to determine whether the reduction in Smo protein levels was directly caused by altered *miR-375* expression (Figure 5D’). Thus, the differential effects of these four miRNAs on Smo protein levels, and sometimes even opposite effects observed for anteriorly and posteriorly localized Smo, suggest that they must regulate Hh signaling through distinct mechanisms.

**FIGURE 7:**
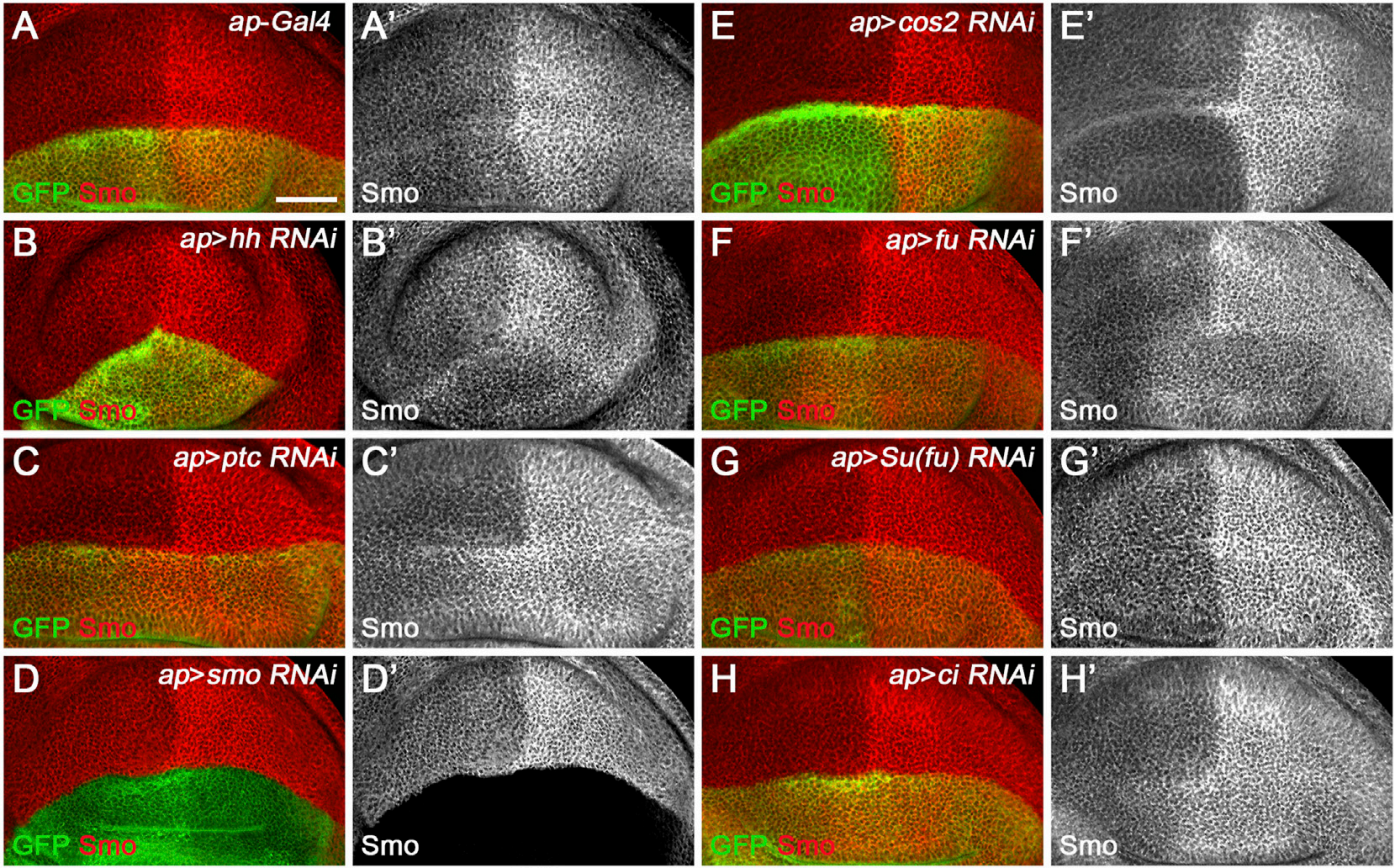
Effects of core Hh pathway gene knockdown on Smo protein levels. Smo expression in wing discs of the indicated genotypes is shown. **(A–B’’)** *ap*-Gal4-driven *hh* RNAi caused a slight decrease in Smo protein levels in both anterior and posterior compartment cells **(B’)**. **(C,C’,H,H’)** Knockdown of either *ptc* or *ci* resulted in a similar increase in Smo protein levels in the cells of the anterior compartment adjacent to the A-P boundary **(C’,H’)**. **(D,D’)** Knockdown of *smo* resulted in loss of Smo expression, as expected **(D’)**. **(E–G’)** Reducing *cos2* or *Su(fu)* expression had no apparent effects on Smo expression **(E’,G’)**. In addition, knockdown of *fu* specifically reduced Smo protein levels in the posterior compartment **(F’)**. Scale bar, 50 μm.

Since *miR-958* had the same effect on Smo expression in the anterior and posterior compartments, it may control Hh signaling at the level of *smo* or its direct regulators. We therefore used *gfp*: *3*′*UTR* ^*smo*^ sensor flies to test whether *smo* was a direct target of *miR-958*. As hypothesized, overexpression of *miR-958* by *ptc*-Gal4 along the A-P boundary resulted in a significant reduction in *gfp*: *3*′*UTR* ^*smo*^ sensor activity (Figures 6D–D”) but not the *gfp*: *3*′*UTRmut* ^*smo*^ control sensor activity (Figures 6E–E”), confirming that *smo* is a direct target of *miR-958*. After examining the effects of *miR-958* on the remaining miRNA sensor flies in our miRNA senor toolbox, we found that *smo* is the only target of *miR-958* in the core components of the Hh signaling cascade (Supplementary Figure S5). For the other three miRNAs that indirectly control Smo protein levels, we found that *miR-133* and *miR-927* did not act on any of the seven sensors in the toolbox (Supplementary Figures S6, S8), while *miR-375* overexpression slightly reduced GFP expression in the wing disc of *gfp: 3*′*UTR* ^*fu*^ sensor flies (Supplementary Figures S7E–E”). Given that no predicted binding sites for *miR-375* was found in the 3′ UTR of *fu*, we speculated that it may regulate the activity of *gfp: 3*′*UTR* ^*fu*^ in an indirect manner. Moreover, we investigated the effect of reducing the expression of seven core components of Hh signaling on Smo protein levels in wing discs to examine whether *miR-133*, *miR-375*, and *miR-927* regulate Smo through these core components of Hh signaling. When *hh* was knocked down by *ap*-Gal4-driven RNAi in the dorsal compartment, downregulation of Smo protein was observed both anteriorly and posteriorly (Figure 7B’). In contrast, reducing Ptc activity by RNAi resulted in elevated Smo levels only in anterior cells (Figure 7C’). As expected, *ap*-Gal4-driven RNAi of smo resulted in ablation of Smo protein (Figure 7D’). Although Smo forms distinct signaling complexes with Cos2, Fu, Su(fu), and Ci, knockdown of *cos2* or *Su(fu)* had no effects on Smo (Figures 7E’,G’). In contrast, reduced *fu* expression resulted in Smo downregulation only in the posterior compartment (Figure 7F’). Surprisingly, we found that knocking down *ci* produced similar effects as *ptc* RNAi (Figure 7H’). However, the above-mentioned phenotypes are not the same as the effects caused by overexpression of *miR-133*, *miR-375*, or *miR-927*, so we believe that the other three miRNAs in the second category may act through targets other than the seven core components of the Hh signaling pathway.

### *miR-190* and *miR-964* Target Genes Other Than the Core Hh Pathway Components

**FIGURE 8:**
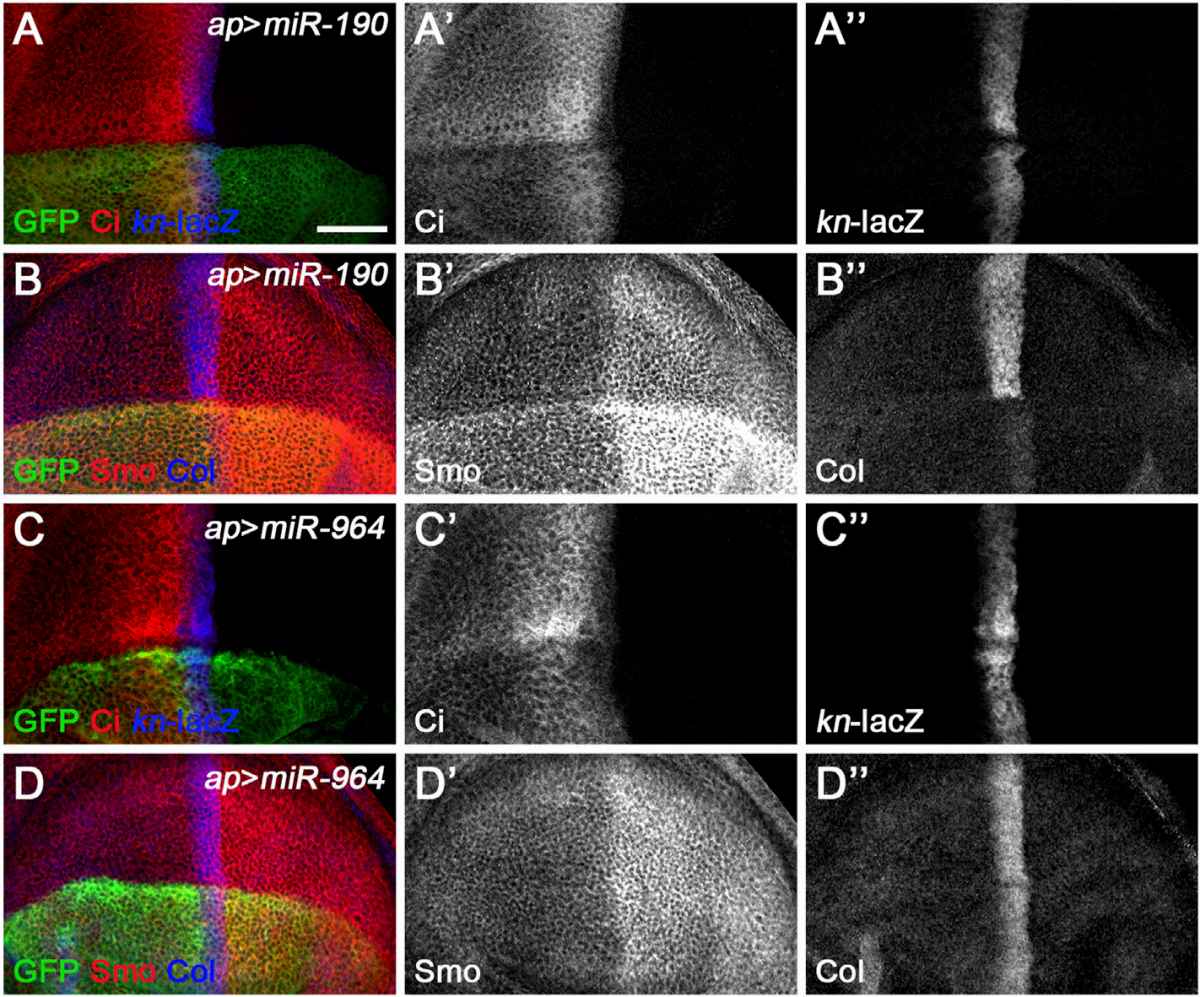
*miR-190* and *miR-964* act as negative regulators of Hh signaling. Ci^FL^ levels and expression of *kn*-lacZ, Smo, and Col in wing discs overexpressing either *miR-190* **(A–B”)** or *miR-964* **(C–D”)** were monitored by immunostaining. Overexpression of *miR-190* or *miR-964* led to obviously reduced levels of Ci^FL^ **(A’,C’)** and almost unchanged *kn*-lacZ activity **(A”,C”)**. Increased *miR-190* expression induced a significant increase in Smo protein levels in both anterior and posterior compartment cells **(B’)** and abrogation of Col expression **(B”)**, whereas overexpressing *miR-964* did not affect the expression of Smo and Col **(D’,D”)**. Scale bar, 50 μm.

A third class of newly discovered Hh signaling-regulating miRNA includes *miR-190* and *miR-964*, since overexpression of either had similar effect, significantly reducing Ci^FL^ levels, while *kn*-lacZ expression was almost unchanged (Figures 8A–A”,C–C”). These phenotypes contrast directly with those observed for class I Hh signaling-regulating miRNA. In addition, their functions for Smo and Col proteins also differ significantly. Overexpression of *miR-964* did not affect the expression of Col or Smo (Figures 8D–D”). This result is similar to loss-of-function *Su*(*fu*) in wing disc cells (Figure 2G’, Figure 7G’), making *Su*(*fu*) a potential target for *miR-964*. We tested this hypothesis using the *gfp*: *3*′*UTR* ^*Su(fu)*^ miRNA sensor flies. However, overexpression of *miR-964* did not alter GFP expression in the *Su*(*fu*) miRNA sensor fly wing disc (Supplementary Figures S9F–F”). This is also consistent with the lack of a *miR-964* binding site in the 3′ UTR of *Su(fu)*. Based on these observations, we propose that *miR-964* negatively regulates Hh signaling and controls Su(fu) activity through unknown target genes.

We noticed a clear difference between the effect of *miR-190* overexpression on *kn*-lacZ activity and the effect on Col protein expression. While the *ap*-Gal4-induced increase in *miR-190* activity in the dorsal compartment had no apparent effects on *kn*-lacZ expression (Figure 8A’’ and Supplementary Figure S10A”), Col protein levels were almost completely abolished compared with wild-type ventral cells (Figure 8B’’ and Supplementary Figure S10A’’’). The presence of six putative *miR-190* binding sites in the 3′ UTR of *col* (Supplementary Figures S10B–E) suggests that *col* may be a target of *miR-190* and that the large reduction in Col expression may be caused by the direct effect of *miR-190* on *col* mRNA. If this is the case, it explains why the *kn*-lacZ expression has not changed. In addition, overexpressed *miR-190* also resulted in a strong increase in Smo expression in both anterior and posterior cells (Figure 8B’), possibly due to the transcriptional upregulation of *smo* in *miR-190*-overexpressing cells. The effect of *miR-190* overexpression on Hh signaling is distinct from the phenotype resulting from loss of any single core component of Hh signaling. Consistently, *miR-190* overexpression did not alter GFP expression in any of the miRNA sensor toolbox flies (Supplementary Figure S11), implying that *miR-190* can regulate Hh signaling at multiple steps through novel Hh signaling players.

### Newly Discovered miRNAs Function in a Cell Autonomous Manner and May Regulate Hh Signaling During Spermatogenesis

**FIGURE 9:**
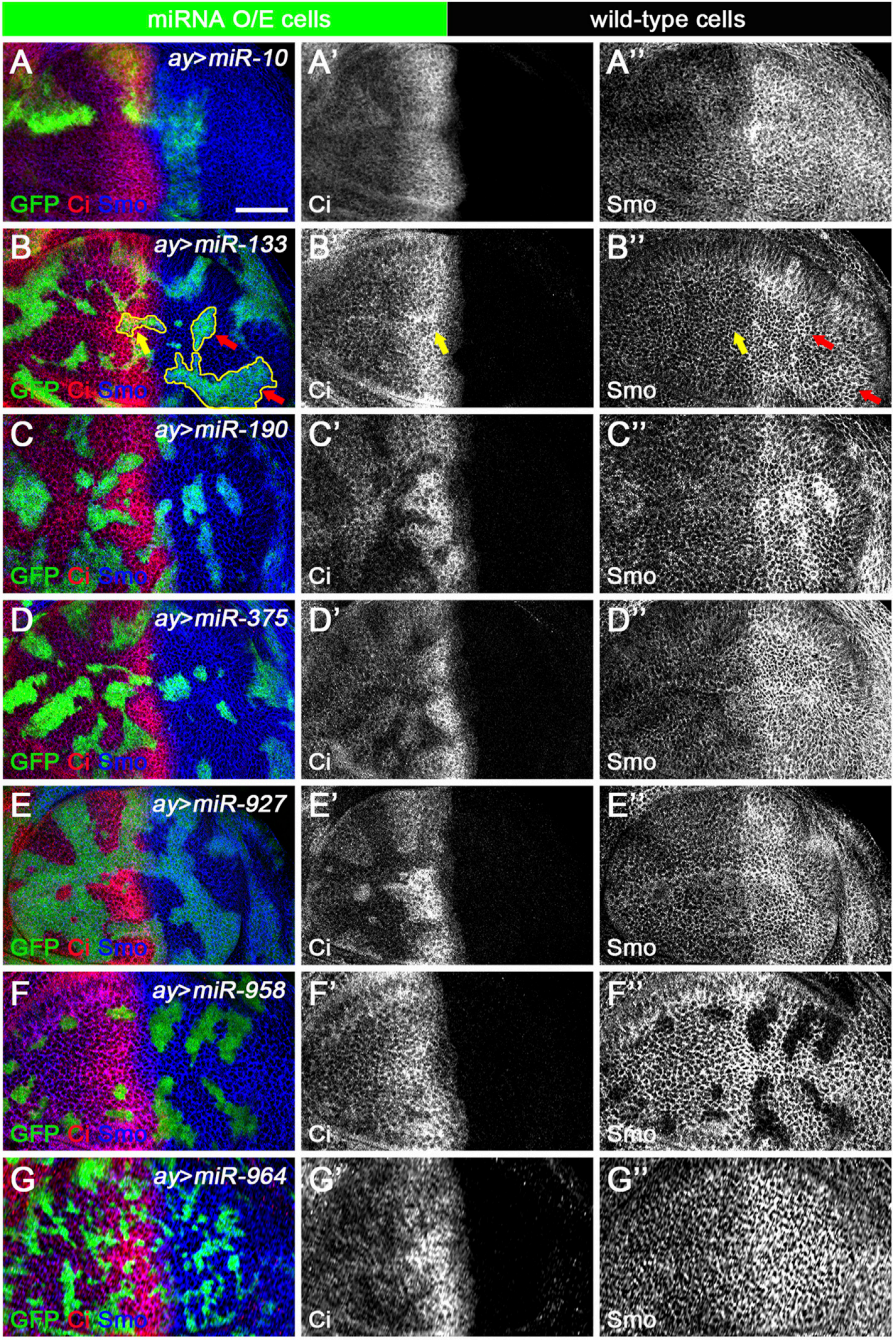
Newly discovered miRNAs regulate Hh signaling in a cell-autonomous manner. All miRNAs were overexpressed in GFP-marked clones in wing discs induced by the FLIPout technique. Ci^FL^ levels and Smo expression were visualized by immunostaining. **(A–A’’)** In *miR-10* overexpressed cells, Ci^FL^ levels were slightly increased **(A’)**, while Smo protein levels were reduced in clones located in the anterior compartment **(A”)**. **(B–B’’)** Ci^FL^ levels were only decreased in *miR-133*-expressing clones adjacent to the A-P boundary **(**yellow arrow in **B’)**, and Smo expression was increased in clones located in the posterior compartment **(**red arrows in **B”)**, but not anterior compartment **(**yellow arrows in **B”)**. **(C–E’’,G–G’’)** Overexpression of *miR-190*, *miR-375*, *miR-927*, and *miR-964* all resulted in a significant decrease in Ci^FL^ levels **(C’,D’,E’,G’)**. However, they had different effects on Smo expression. Overexpression of *miR-190* led to increased Smo levels in anterior and posterior clones **(C”)**, whereas increased expression of *miR-375* resulted in decreased Smo levels, especially in anterior clones **(D”)**. Overexpression of *miR-927* or *miR-964* had no apparent effects on Smo expression **(E”,G”)**. **(F–F’’)** Increased expression of *miR-958* led to a mild decrease in Ci^FL^ levels in clones located in the anterior compartment, whereas Smo expression was completely lost in clones located in both anterior and posterior compartments **(F”)**. Scale bar, 50 μm.

The *ap*-Gal4 driver was used to efficiently induce miRNA overexpression in the dorsal compartment of the wing disc. However, in some cases, increased miRNA expression resulted in patterning defects that prevented us from clearly discerning their functions. This was the case for *miR-375* (Figures 5D–D”). To overcome this deficiency, we used the FLIPout technique (Ito et al., 1997; Pignoni and Zipursky, 1997) to induce *miR-375* overexpression in only a few wing epithelial cells and found that FLIPout clones had significantly reduced Smo protein in the anterior compartment of the wing disc (Figures 9D–D”). The inability of *miR-375* to reduce Smo protein levels in posteriorly localized FLIPout clones suggests that *miR-375* does not control *smo* transcription, but rather functions in a step after Hh activation. The above FLIPout analysis indicates that *miR-375* regulates Hh signaling in a cell-autonomous manner. This also applies to the remaining six miRNAs (Figures 9A–C”,E–G”). However, one difference was noticed. Unlike *ap*-Gal4-induced *miR-133* activity (Figure 5A’), Ci and Smo protein levels were selectively affected in *miR-133* FLIPout wing disc clones (Figures 9B–B”). This inconsistency may be due to different timings used to induce *miR-133* expression.

**FIGURE 10:**
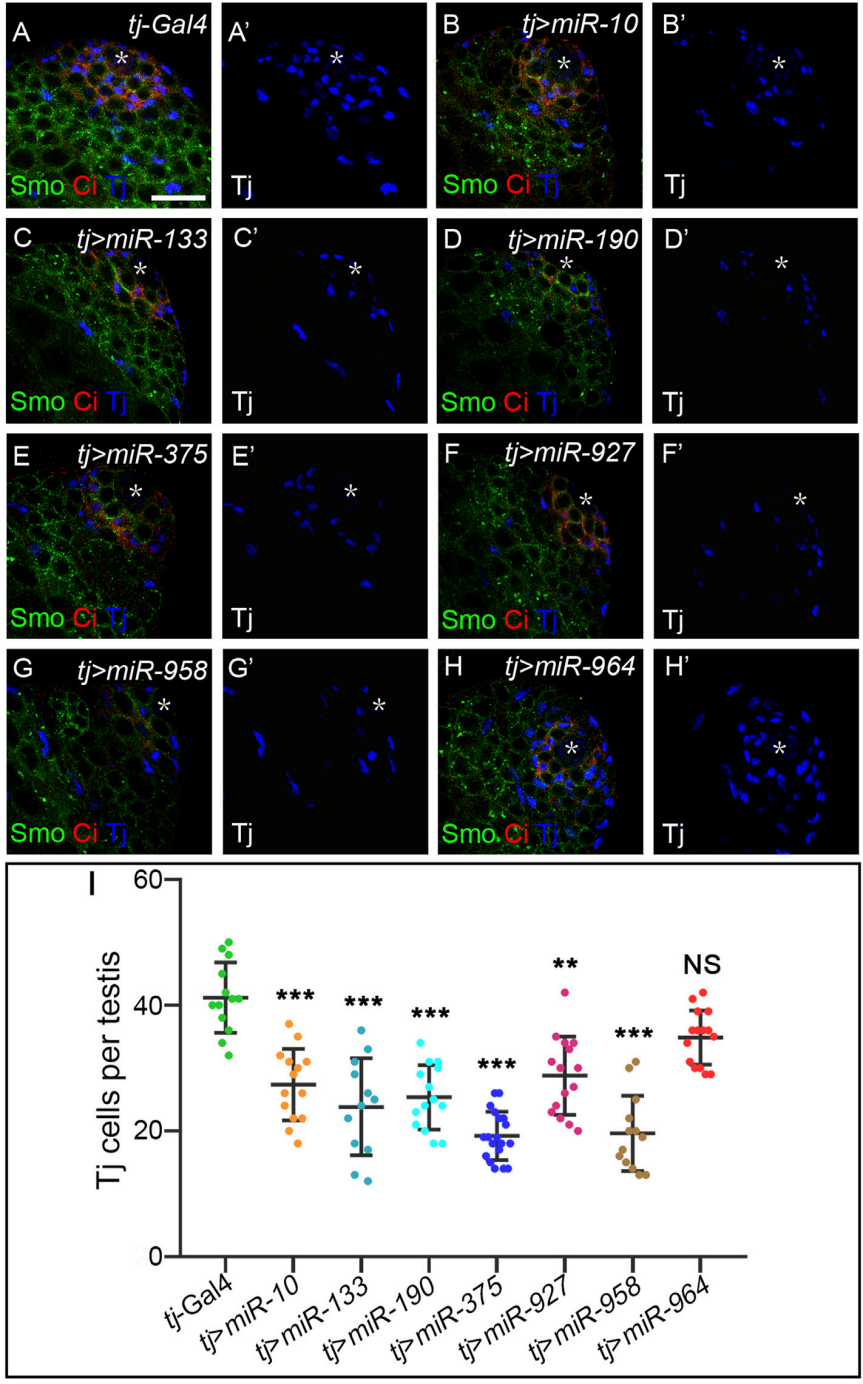
Newly discovered miRNAs, except *miR-964*, are involved in Hh signaling-controlled CySC maintenance during spermatogenesis. **(A–H”)** All newly discovered miRNAs were specifically overexpressed by *tj*-Gal4 in early cyst cells. Ci^FL^ levels and expression of Smo and Tj were monitored by immunostaining. Overexpression of *miR-10*, *miR-133*, *miR-190*, *miR-375*, *miR-927*, and *miR-958*, but not *miR-964*, obviously reduced the number of Tj-positive cells in the testis. **(I)** Shown is statistical analysis of the number of Tj-positive cells per testis. Data are presented as mean ± S.D. (n^*tj-Gal4*^ = 13, n^*tj>miR−10*^ = 14, n^*tj>miR−133*^ = 12, n^*tj>miR−190*^ = 14, n^*tj>miR−375*^ = 19, n^*tj>miR−927*^ = 15, n^*tj>miR−958*^ = 13, n^*tj>miR−964*^ = 15). One-way ANOVA followed by Dunnett’s tests was used. NS, not significant. *p < 0.05. **p < 0.01. ***p < 0.001. Scale bar, 25 μm.

Hh morphogens function in a cell-nonautonomous manner to regulate a range of developmental events beyond wing development (Guerrero and Kornberg, 2014). One of the systems that best illustrates Hh-mediated cell specification is *Drosophila* testis development. Hh is produced in niche cells in the apical tip of the testis and activates Hh signaling in adjacent cyst stem cells (CySCs) to maintain their self-renewal. As a result, Ci^FL^ and Smo proteins are found to be more stable around the niche (Michel et al., 2012; Amoyel et al., 2013). The transcription factor Traffic jam (Tj) marks CySCs and early cyst cells (Li et al., 2003). When Hh signaling activity is impaired, the number of Tj-positive cells is significantly reduced (Michel et al., 2012; Amoyel et al., 2013; Zhang et al., 2013b). Conversely, hyperactivation of Hh signaling in somatic cells leads to massive overproduction of CySCs (Zhang et al., 2013b). We used the *tj*-Gal4 driver to specifically overexpress our newly discovered miRNAs in somatic cells and examined their effect on the number of Tj-positive cyst cells. Our results showed that overexpression of each miRNA, except *miR-964*, resulted in a 24%–48% reduction in the number of Tj-positive cells (Figure 10), suggesting that these miRNAs negatively regulate Hh signaling to maintain cyst cell homeostasis. We noted that induction of *miR-964* activity in cyst cells had no effects, consistent with its regulation of *Su*(*fu*). Taken together, the above results suggest that the newly discovered miRNAs, when overexpressed in cyst cells, can regulate Hh signaling during spermatogenesis and may act as general negative regulators of Hh signaling.

### Loss-Of-Function Analysis of the Newly Discovered miRNAs

To better understand the endogenous functions of the newly discovered miRNAs, we obtained and analyzed knockout lines for all seven miRNAs (Chen et al., 2014). We found that *miR-190* ^*KO*^ was lethal at the third instar larval stage, while the remaining six miRNA mutants survived into adulthood. These features allowed us to study the effect of individual deletions of these miRNAs on the transcription of Hh signaling components (*hh*, *ptc*, *smo*, *cos2*, *fu*, *Su(fu)*, *ci*, *ihog*, *boi*, *dally*, and *dally-like* (*dlp*)), and downstream targets (*col* and *dpp*) as well as Ci^FL^, Col and Smo protein levels. Furthermore, the Hh signaling-related phenotypes of adult wing morphogenesis of these miRNA knockout mutants were also examined.

**FIGURE 11:**
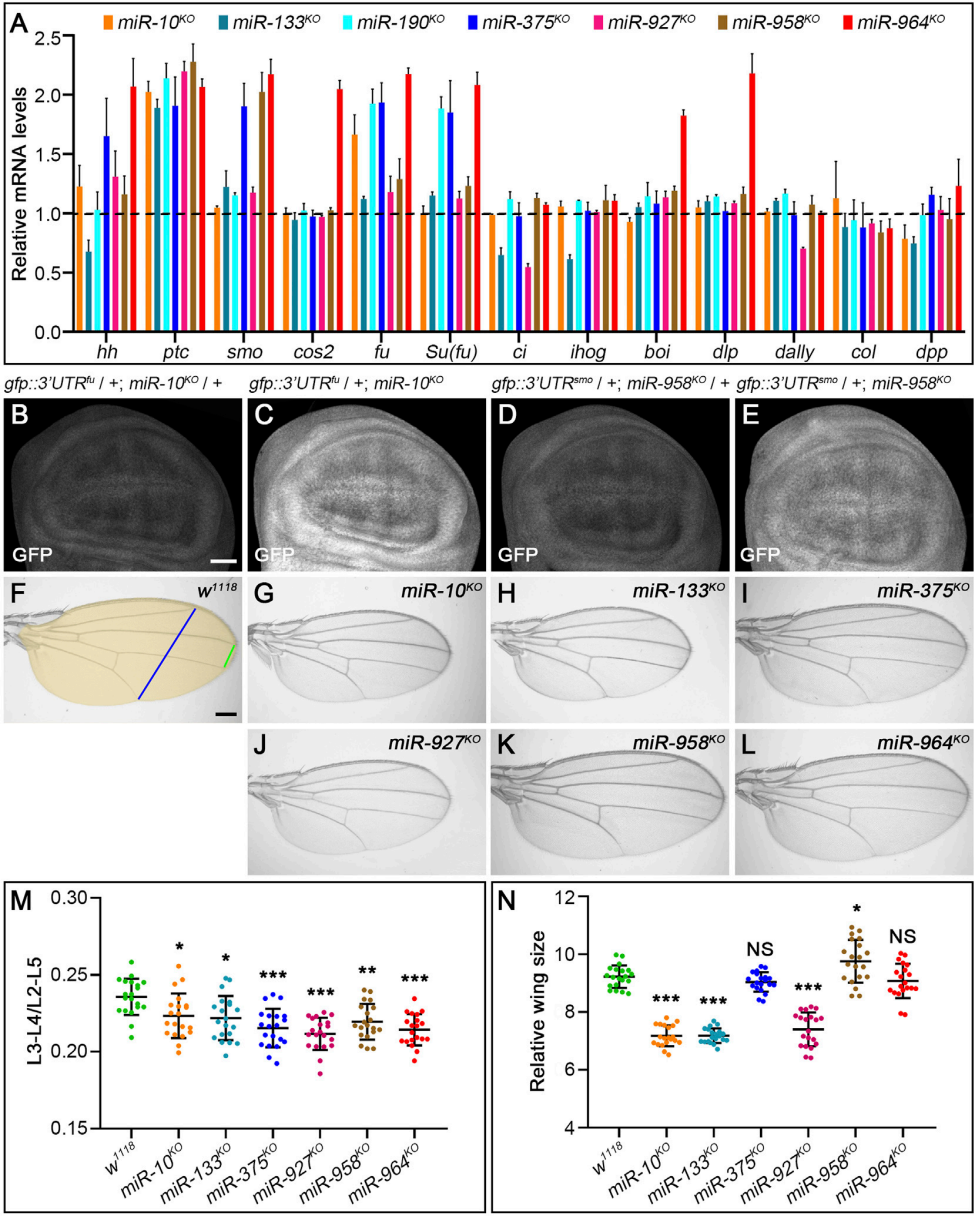
Loss-of-function analysis of the seven newly discovered miRNAs. **(A)** Quantification of mRNA expression of *hh*, *ptc*, *smo*, *cos2*, *fu*, *Su(fu)*, *ci*, *ihog*, *boi*, *dally*, *dlp*, *col*, and *dpp* in seven miRNA knockout mutant larvae by qPCR. Bar plots represent relative mRNA levels of indicated genotypes (*n* = 3); error bars represent standard deviation (S.D.). **(B–E)** The GFP expression of the *gfp*: *3’UTR* ^*fu*^ sensor was significantly increased in *miR-10* ^*KO*^ homozygotes **(C)** compared with *miR-10* ^*KO*^ heterozygotes **(B)**. The same goes for the *gfp*:*3’UTR* ^*smo*^ sensor in *miR-958* ^*KO*^ homozygotes **(D,E)**. **(F–L)** Adult wings of the indicated genotypes are shown. The distance between L3-L4 veins **(**green line in **F)**, the distance between L2-L5 veins **(**blue line in **F)**, and the size of adult wings **(**yellow area in **F)** were measured, **(M,N)** Shown is statistical analysis of the ratio of L3-L4 distance to L2-L5 distance **(M)**, and wing size **(N)** for the indicated genotypes. Data are presented as mean ± S.D. (*n* = 20). One-way ANOVA followed by Dunnett’s tests was used. NS, not significant. *p < 0.05. **p < 0.01. ***p < 0.001. Scale bar, **(B–E)**, 50 μm; F-L, 100 μm.

**FIGURE 12:**
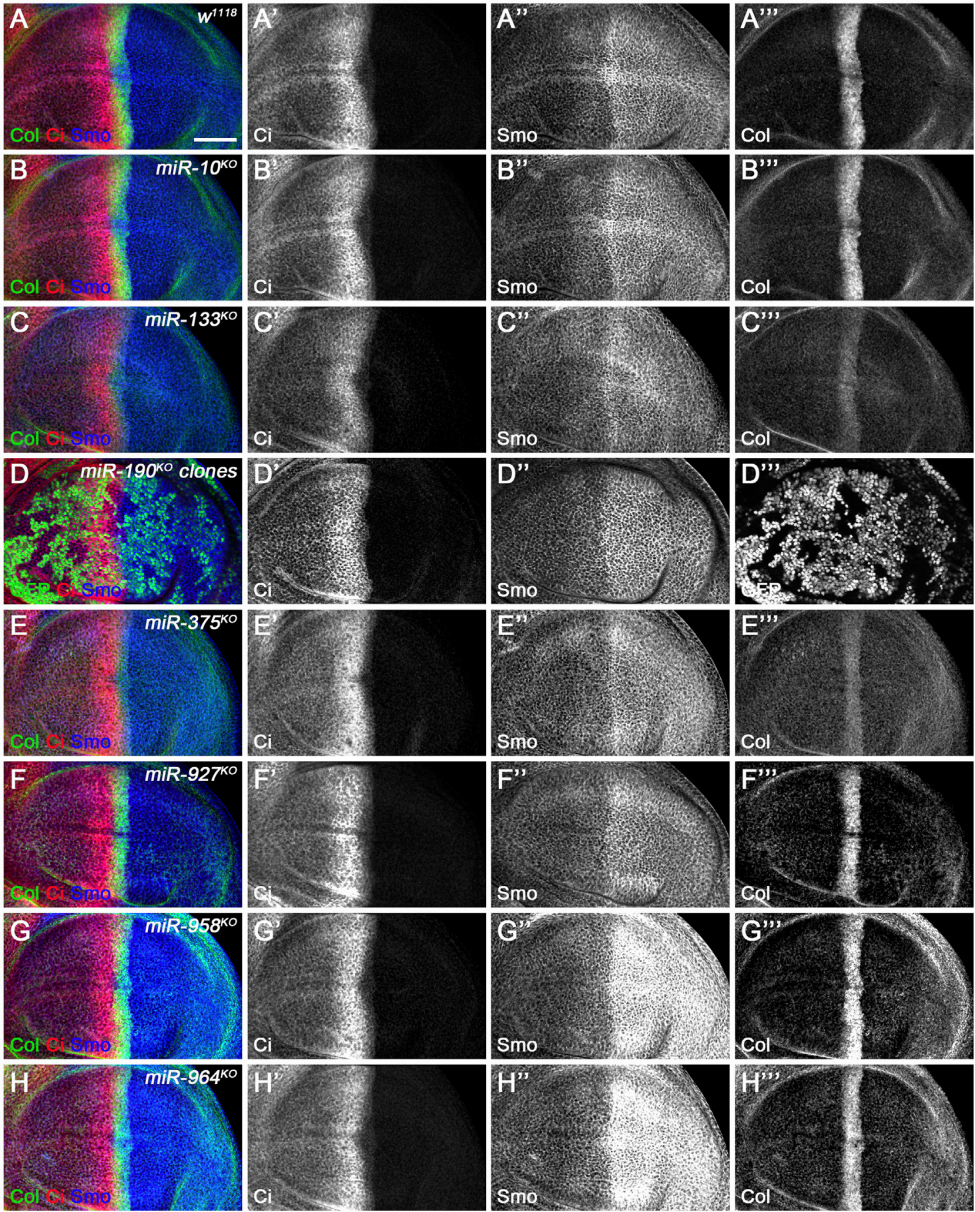
Ci^FL^ and Smo levels and Col expression in loss-of-function mutants of newly discovered miRNAs. Ci^FL^ and Smo protein levels and Col expression were visualized by immunostaining in third instar larval wing discs of the indicated genotypes. Loss of *miR-10* or *miR-927* had no apparent effects on Ci^FL^ and Smo levels and Col expression **(B–B’’’, F–F’’’)**. Ci^FL^ levels and Col expression were significantly reduced in *miR-133* ^*KO*^ mutants **(C’,C’’’)**, while Smo levels remained unchanged **(C”)**. Ci^FL^ and Smo levels were unaffected in *miR-190* ^*KO*^ clones negatively marked by GFP **(D–D’’’)**. Col expression was significantly decreased in *miR-375* ^*KO*^ mutants **(E’’’)**, whereas Ci^FL^ and Smo levels were unchanged **(E’,E”)**. Deletion of *miR-958* or *miR-964* significantly elevated Smo levels in the posterior compartment **(G”,H”)**, but hardly changed Ci^FL^ levels and Col expression **(G’,G’’’,H’,H’’’)**. Scale bar, 50 μm.

As expected, transcripts of *fu* and *smo* were significantly upregulated in *miR-10* ^*KO*^ and *miR-958* ^*KO*^ mutants, respectively (Figure 11A). Consistently, the activities of miRNA sensors for *fu* and *smo* were respectively enhanced in *miR-10* ^*KO*^ and *miR-958* ^*KO*^ wing discs (Figures 11B–E). Since overexpression of *fu* had no visible effects on adult wing morphology and Hh signaling activity (Claret et al., 2007), it is not surprising that Ci^FL^, Smo and Col protein levels were not altered in *miR-10* ^*KO*^ mutant wing discs (Figures 12B–B”). In *miR-10* ^*KO*^ mutants, however, adult wings were significantly reduced in size (Figures 11G,N), likely due to the effect of additional *miR-10* targets important for wing development.

The upregulation of *smo* transcripts in the *miR-958* ^*KO*^ mutant was confirmed at the protein level, as obviously increased Smo protein levels were observed in the posterior compartment of the wing disc (Figure 12G’’). In contrast, anteriorly localized Smo was only slightly upregulated (Figure 12G’’). This differential effect of *miR-958* ^*KO*^ on Smo protein can be attributed to post-translational regulation of Smo protein stability in anterior wing disc cells (Denef et al., 2000). Previously studies have shown that overexpression of wild-type Smo is not sufficient to induce a distinct gain-of-function Hh signaling phenotype (Hooper, 2003; Zhu et al., 2003). Thus, only the Iroquois (Iro) expression domain, the Hh target representing low-level Hh activity, is expanded, but not the medium-to-high Hh signaling targets Dpp, Ptc, and Col (Hooper, 2003). Consistent with these reports, the adult wing size of *miR-958* ^*KO*^ mutant flies increased only slightly (Figures 11K,N), while the levels of Ci^FL^ and Col in the wing disc remained unchanged (Figures 12G’,G’’’). In addition to *miR-958*, significantly elevated Smo were observed in the posterior compartment of *miR-964* ^*KO*^ wing discs (Figure 12H’’). Unlike *miR-958*, the mRNA levels of *hh*, *smo*, *cos2*, *fu*, *Su(fu)*, *boi*, and *dlp* were all increased in *miR-964* ^*KO*^ larvae (Figure 11A).

For the remaining four newly discovered miRNAs, we found reduced adult wing size in *miR-133* ^*KO*^ and *miR-927* ^*KO*^ flies (Figures 11H,J,N). This phenotype is very consistent with marked reductions in Ci^FL^ stability and Col expression (Figures 12C’,C’’’) and reduced *ci* and *hh* transcription in *miR-133* ^*KO*^ mutants (Figure 11A). Likewise, decreased expression of *ci* and *dally* was observed in *miR-927* ^*KO*^ larvae (Figure 11A). While *fu* and *Su(fu)* expression was increased in *miR-190^KO^* and *miR-375^KO^* mutant larvae (Figure 11A), Smo and Ci^FL^ levels were not changed in *miR-190^KO^* clones (Figures 12D–D’’’). Additional effects were observed in *miR-375^KO^* flies, including decreased Col protein levels (Figure 12E’’’) and *hh* and *smo* expression (Figure 11A). Interestingly, *ptc* expression was increased in all miRNA knockout mutant larvae (Figure 11A), which could partly explain the slight but consistent decrease in the distance between L3-L4 longitudinal veins in adult wing blades (Figure 11M).

Take together, we provide genetic and molecular evidence that endogenous *miR-10* and *miR-958* paly a role in Hh signaling, directly targeting *fu* and *smo*, respectively. We further demonstrate that the loss of the remaining five newly discovered miRNAs results in dysregulated gene expression associated with Hh signaling, suggesting that these miRNAs are required for the maintenance of Hh signaling homeostasis in development.

## Discussion

In this study, we systemically assessed the impact of 179 miRNAs on Hh signaling through an *in vivo* miRNA overexpression screen in *Drosophila* and identified seven miRNAs as novel negative regulators of Hh signaling. We further demonstrated that two of these miRNAs, *miR-10* and *miR-958*, target *fu* and *smo*, respectively, while the other five miRNAs control Hh signaling through targets other than core Hh pathway components. Importantly, loss-of-function analysis indicated that these seven newly discovered miRNAs also regulate Hh signaling *in vivo*.

Prior to our *in vivo* genome-wide screen, another comprehensive miRNA overexpression screen was performed in cultured S2R^+^ cells (Kim et al., 2014). Both screens identified the same set of Hh signaling-regulating miRNAs, including *miR-7*, *miR-10*, *miR-12*, *miR-14*, *miR-133*, *miR-927, miR-932*, and *miR-964*. Furthermore, we discovered two other miRNAs, *miR-190* and *miR-375*, that target as yet unidentified novel Hh signaling regulators in addition to the canonical Hh signaling components. It is not surprising that *miR-190* and *miR-375* were not found in the *in vitro* screen, as the screen was mainly based on changes in the activity of miRNA sensors for known regulators of Hh signaling (Kim et al., 2014). It is also possible that these *in vitro* miRNA sensors expressed in S2R^+^ cells may not be sensitive enough as it did not respond to the *smo*-targeting *miR-958* that emerged from our screen. Furthermore, the activity of the *in vitro* sensors tested in S2R^+^ cells may not reflect Hh regulation as faithfully as in wing epithelial cells. *miR-927* and *miR-964* have been reported to target the 3’ UTR of *ci* in S2R^+^ cells (Kim et al., 2014). When overexpressed, these two miRNAs had no effects on GFP expression in the *ci* miRNA sensor flies. *miR-133*, another positive result from our *in vivo* screen, was thought to target *CK1α* and *GSK3* (Kim et al., 2014), which encode two negative regulators of Hh signaling (Price and Kalderon, 2002; Su et al., 2011). However, increased *miR-133* expression in wing discs did not upregulate Hh signaling, but significantly decreased Hh signaling by reducing Ci and Col protein levels, strongly suggesting that *CK1α* and *GSK3* are not direct targets of *miR-133 in vivo.*

Among seven novel miRNAs involved in Hh signaling *in vivo*, we found that *miR-10* and *miR-958* target *fu* and *smo*, respectively, while the direct targets of the other five miRNAs, *miR-133*, *miR-190*, *miR-375*, *miR-927*, and *miR-964*, have not been identified from the Hh signaling regulatory network. Nevertheless, direct targets of some of these miRNAs have been reported experimentally in *Drosophila,* and they play important roles in processes other than Hh signaling. For example, *miR-133* targets the phosphodiesterase encoding *Pde1c* to regulate epithelial-mesenchymal transition in wing discs (Jung et al., 2021). *fga* is a direct target of *miR-190* and inhibits the HIF-dependent hypoxia response (De Lella Ezcurra et al., 2016). *miR-927* controls larval growth through its target *Kr-h1* (He Q. et al., 2020), while *miR-964* targets *Drs* to inhibit Toll signaling in response to bacterial infection (Li et al., 2017). Among these validated targets, *Pde1c* may be involved in Hh signaling regulation, as the phosphodiesterase family protein PDE4D is known to enhance Hh signaling activity by inhibiting PKA in human medulloblastoma cells (Ge et al., 2015). Other targets did not show modulating Hh signaling. Further experiments are required to determine their potential role in Hh signaling.

It should be noted that the validated targets of *miR-10* and *miR-958* in our study, *fu* and *smo*, as well as the targets of the other five miRNAs mentioned above, are listed in the miRNA target prediction database TargetScan (http://www.targetscan.org/). For the five miRNAs whose *bona fide* targets for Hh signaling have not yet been identified, further analysis of the predicted target set for each miRNA in TargetScan may expand the miRNA network that regulates Hh signaling. *Synaptobrevin* (*Syb*), another predicted target of *miR-133*, is required for basal cytoneme formation. Hh gradient and signaling activity are impaired when *Syb* is knocked down by RNAi (Chen et al., 2017). *tout-velu* (*ttv*) is a predicted target gene of *miR-190*. It encodes a glycosyltransferase that positively regulates Hh signaling through its role in the biosynthesis of heparan sulfate proteoglycans (HSPGs), which are required for Hh morphogen propagation. In *ttv* mutant clones, both Ci^FL^ levels and expression of downstream targets of Hh signaling, such as *ptc*, are reduced (Bellaiche et al., 1998; Bornemann et al., 2004). As Ci^FL^ levels and Col expression were significantly decreased in *miR-190*-overexpressing cells (Figures 8A’,B” and Figure 9C’), *miR-190* could regulate Hh signaling by targeting *ttv*. In addition, *brother of tout-velu* (*botv*), another glycosyltransferase-encoding gene required for the spread of the Hh morphogen (Han et al., 2004; Takei et al., 2004), is a predicted target of *miR-375*. Similar to *ttv*, Hh signaling is significantly impaired in *botv* mutant clones, resembling the *miR-375* overexpression phenotype. Therefore, *botv* may be a potential target of *miR-375* to regulate Hh signaling. Interestingly, no predicted target genes of *miR-927* and *miR-964* were reported to play a role in Hh signaling, suggesting that these two miRNAs may regulate Hh signaling by targeting novel players in the Hh regulatory network.

In addition to the TargetScan database, a recent study provided a new set of binding sites for the top 59 CLIP-enriched miRNAs by assigning miRNA seed matches on PAR-CLIP and HITS-CLIP of Argonaute-1 (AGO1) (Wessels et al., 2019). According to this study, *miR-190* has 323 predicted targets (Supplementary Table S3), of which seven genes are involved in Hh signaling, namely *crooked neck* (*crn*), *Multi drug resistance 49* (*Mdr49*), *brother of odd with entrails limited* (*bowl*), *Cullin1* (*Cul1*), *Histone deacetylase 1* (*HDAC1*), *G protein-coupled receptor kinase 1* (*Gprk1*), and *Gprk2* (Ou et al., 2002; Kent et al., 2006; Benítez et al., 2009; Cheng et al., 2010; Zhang et al., 2013a; Liu et al., 2014; Deshpande et al., 2016). Among them, Crn, Mdr49, and Bowl are positive regulators of Hh signaling. Given the negative role of *miR-190* in Hh signaling, it may regulate Hh signaling by targeting these three candidate genes.

Since miRNAs contribute to many gene regulatory networks and diverse signaling pathways, it is not surprising that they play important roles in multisteps of cancer development, including cell proliferation, apoptosis, metastasis, and angiogenesis (Lee and Dutta, 2009; Bartel, 2018; He B. et al., 2020). In our screen, four newly discovered miRNAs are conserved from *Drosophila* to humans and have been implicated in the development of various cancers. However, the targets of some of these miRNAs are only found in certain types of cancer. For example, *miR-10* regulates the oncogene *USF2* in myeloid leukaemia (Agirre et al., 2008; Jongen-Lavrencic et al., 2008), *BDNF* in cervical cancer (Zhai et al., 2017), and *Tiam1* in gastric cancer (Liu F. et al., 2021). Likewise, *miR-190* acts through multiple targets and exerts its tumor suppressor effect in various cancer types, including breast, colon and prostate cancer, glioma, and hepatocellular carcinoma (Yu and Cao, 2019). However, the targets of *miR-10* in intestinal neoplasia (Stadthagen et al., 2013) and *miR-190* in cervical and rectal cancer remain unknown (Yu and Cao, 2019). As for *miR-133* and *miR-375*, two other conserved miRNAs, their potential underlying regulatory mechanisms in their respective cancers and their true targets of action remain unclear and require further study (Bandrés et al., 2006; Arvidsson et al., 2018). Given that uncontrolled Hh signaling is associated with more than 20% of all forms of cancer (Jeng et al., 2020; Sigafoos et al., 2021), the miRNAs identified in our screen as negative regulators of Hh signaling, most likely control cancer progression through its targeting in the Hh regulatory network. Identifying the *bona fid*e targets of these conserved miRNAs in Hh signaling, especially in *Drosophila* with robust genetics, will greatly aid in the discovery of new therapeutic targets in cancer therapy.

## Materials and Methods

### Fly Genetics

The transgenic miRNA-overexpression and miRNA-knockout fly strains used in this study are listed in Supplementary Table S1. All fly crosses were maintained at 25°C except those listed in Supplementary Table S4. *w* ^*1118*^, *ap*-Gal4 (bl-3041), *dpp*-Gal4, *ptc*-Gal4, UAS-*ptc RNAi* (bl-28795), and UAS-*ci RNAi* (bl-28984) were obtained from the Bloomington *Drosophila* Stock Center. UAS-*cos2 RNAi* (KK#108914), UAS-*fu RNAi* (GD#27662), and UAS-*smo RNAi* (GD#9542) were obtained from the Vienna *Drosophila* RNAi Center. *UAS-hh RNAi* (TH201500473.S) and UAS-*Su(fu) RNAi* (THU3468) were obtained from the TsingHua Fly Center. *tj*-Gal4 was a gift of Zhaohui Wang. *hs*-*flp* ^*122*^; *Act5C* > *yw* > Gal4, UAS-*gfp* was a gift of Haiyun Song. The phenotypes induced by Hh pathway gene knockdown or miRNA overexpression in this study were fully penetrant.

### Immunofluorescence Staining

Wing disc immunofluorescence staining was performed using standard procedures (Su et al., 2011). Testes were immunostained using the described protocol (Inaba et al., 2015). Briefly, adult testes on day 3 post-eclosion were dissected in PBS and fixed in 4% formaldehyde in PBS for 30 min. After permeabilization in 0.3% PBST (PBS + 0.3% Triton X-100) for 1 h the testes were incubated with primary antibody in 3% bovine serum albumin (BSA) in PBST overnight at 4°C. Then, the samples were washed three times in PBST for 20 min each and incubated with secondary antibody in 3% BSA in PBST for 3 h at room temperature. The following primary antibodies were used: mouse anti *β*-galactosidase [1:200; 40-1A; Developmental Studies Hybridoma Bank (DSHB)], rat anti-Ci (1:20; 2A1; DSHB), mouse anti-Smo (1:20; 20C6; DSHB), and guinea-pig anti-Tj (1:5000; a gift of Dorothea Godt) (Gunawan et al., 2013). Alexa Fluor-conjugated secondary antibodies generated in goat (1:400; Invitrogen) were used.

### Antibody Production

A rabbit polyclonal antibody against Col was generated in this study. The full-length Col protein fused with GST was purified and injected into rabbits for immunization, and the sera were further affinity purified to obtain the final antibody (Abclonal Biotech.). This antibody was used for immunostaining at 1:4000.

### Generation of the *in vivo* miRNA Sensors

For φC31 integrase-mediated site-directed integration, the attB sequence was introduced into *pCaSpeR-tub-egfp* (a gift of Xinhua Lin) (Brennecke et al., 2003). The 3′ UTRs of *hh*, *ptc*, *smo*, *cos2*, *fu*, *Su(fu)*, and *ci* were amplified from genomic DNA using the primers listed in Supplementary Table S5, and then cloned into the *pCaSpeR-tub-egfp-attB* plasmid. All miRNA sensor constructs were integrated to the *attP40* site by φC31 integrase. For the convenience of the cross scheme, miRNA sensor lines for *smo* integrated to the *attP2* site were also generated. To generate miRNA-binding sites mutated sensors for *fu* and *smo*, site-directed mutagenesis was performed using a PCR-based approach with primers listed in Supplementary Table S5.

### RNA Isolation and Quantitative Real-Time RT-PCR

Total RNA was extracted from second or third instar larvae using Eastep Super Total RNA Extraction Kit (LS1040; Promega). Reverse transcription was performed with Eastep RT Master Mix Kit (LS 2050; Promega). cDNA levels were quantified by real-time PCR in a 7500 Real-Time PCR System (Applied Biosystems) using 2x Universal SYBR Green Fast qPCR Mix (RM21203; ABclonal). Relative fold changes of *hh*, *ptc*, *smo*, *cos2*, *fu*, *Su(fu)*, *ci*, *ihog*, *boi*, *dally*, *dlp*, *col*, and *dpp* transcripts were calculated using the comparative CT method. Samples from three independent experiments were prepared and run in duplicate. Primers used for qPCR are listed in Supplementary Table S6.

### Quantification and Statistical Analysis

The number of Tj-positive somatic cells in each testis, the ratio of the distance between the L3-L4 longitudinal veins to the distance between L2-L5, and the size in each adult wing of different genotypes were quantified and statistically analyzed using Graphpad Prism 8. One-way ANOVA followed by Dunnett’s test was used. Standard errors of mean were represented. NS, not significant. *p < 0.05. **p < 0.01. ***p < 0.001.

## Data Availability

The original contributions presented in the study are included in the article/Supplementary Material, further inquiries can be directed to the corresponding authors.
