# Supplementary figures and images for: Dissection of the microRNA Network Regulating Hedgehog Signaling in Drosophila
Source: Front Cell Dev Biol. 2022 Apr 28;10:866491. doi: 10.3389/fcell.2022.866491 (PMC9096565; doi:10.3389/fcell.2022.866491)

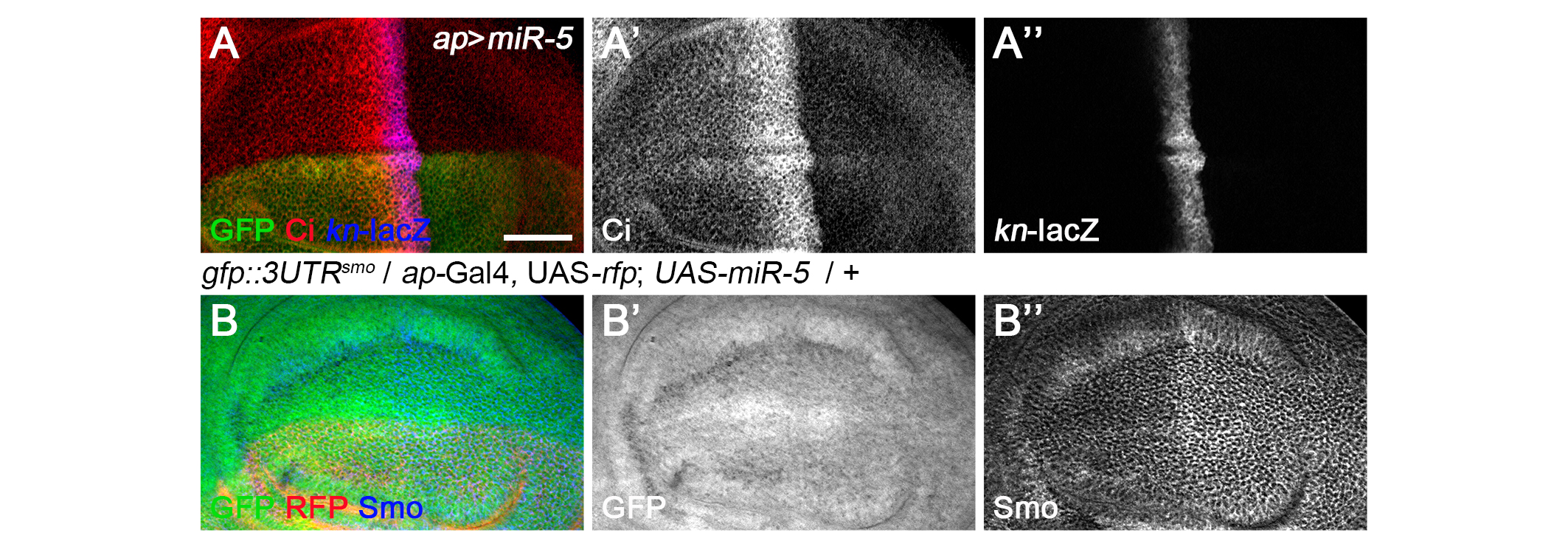

Supplement: Supplementary file 1 [file Image3.JPEG]

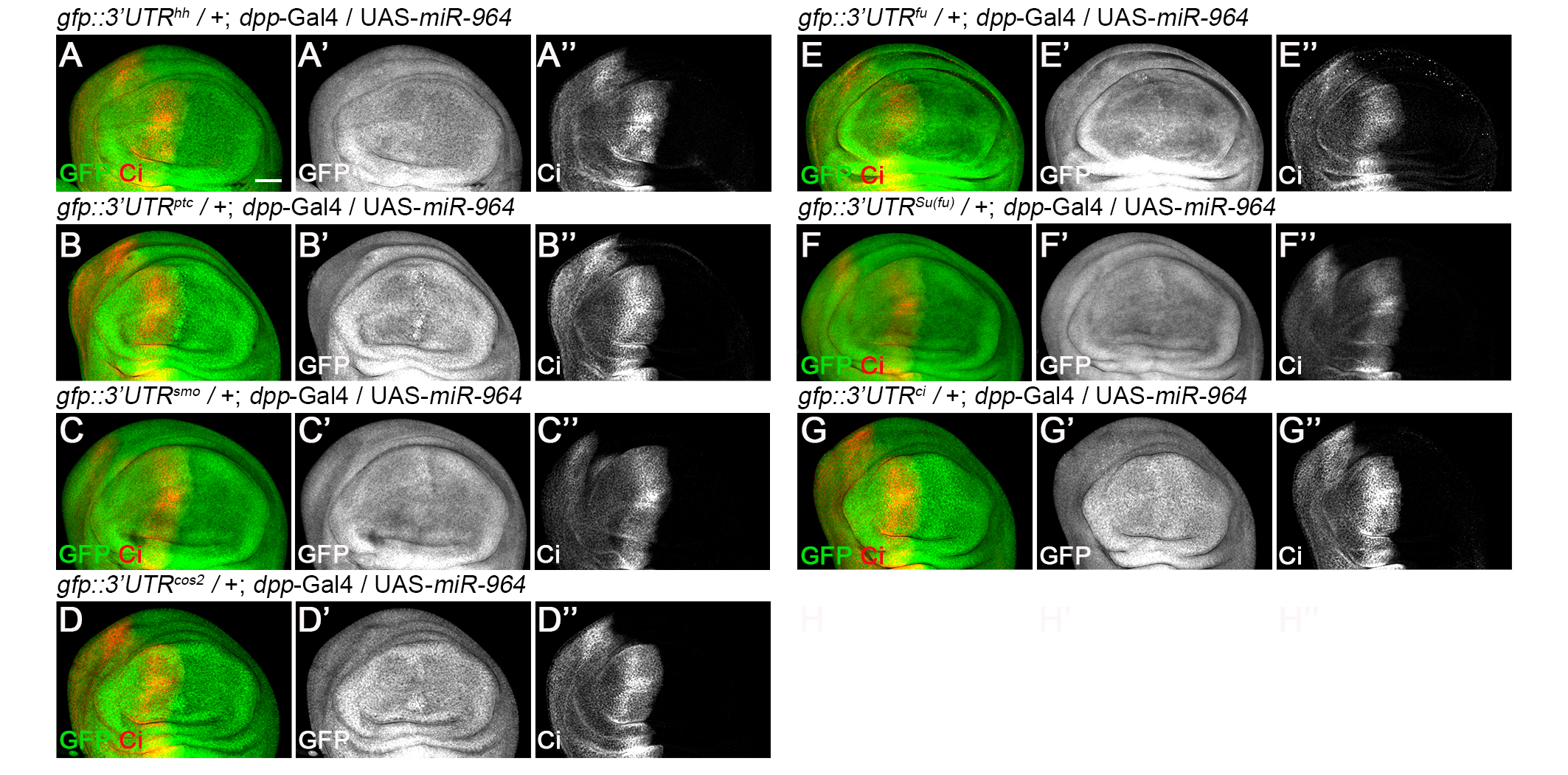

Supplement: Supplementary file 4 [file Image9.JPEG]

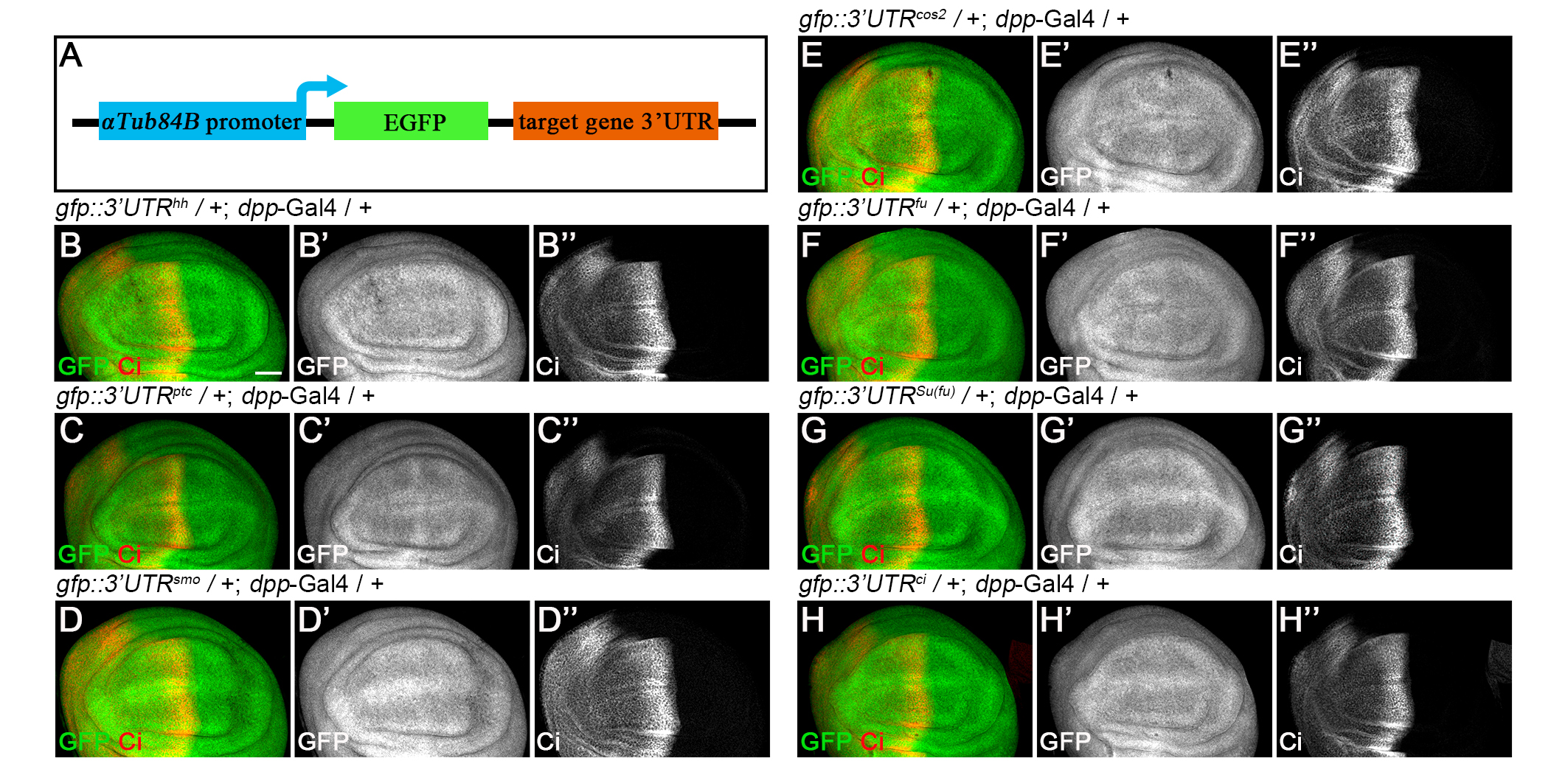

Supplement: Supplementary file 5 [file Image1.JPEG]

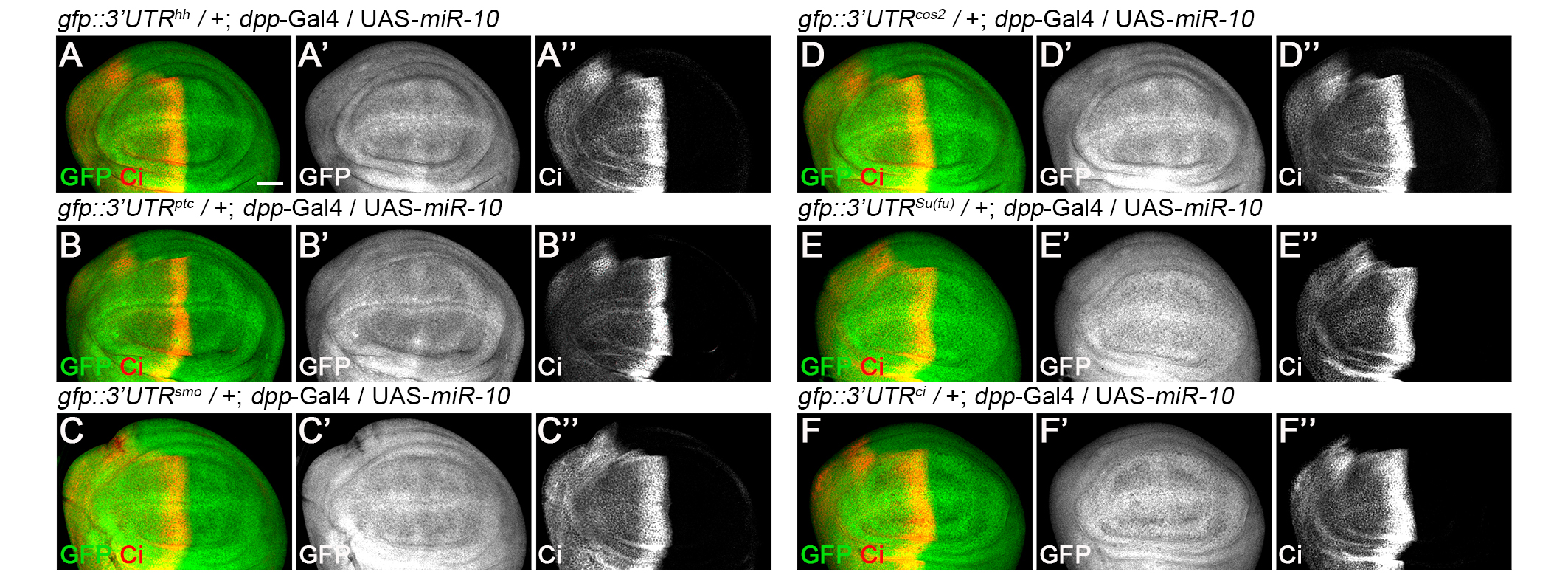

Supplement: Supplementary file 6 [file Image4.JPEG]

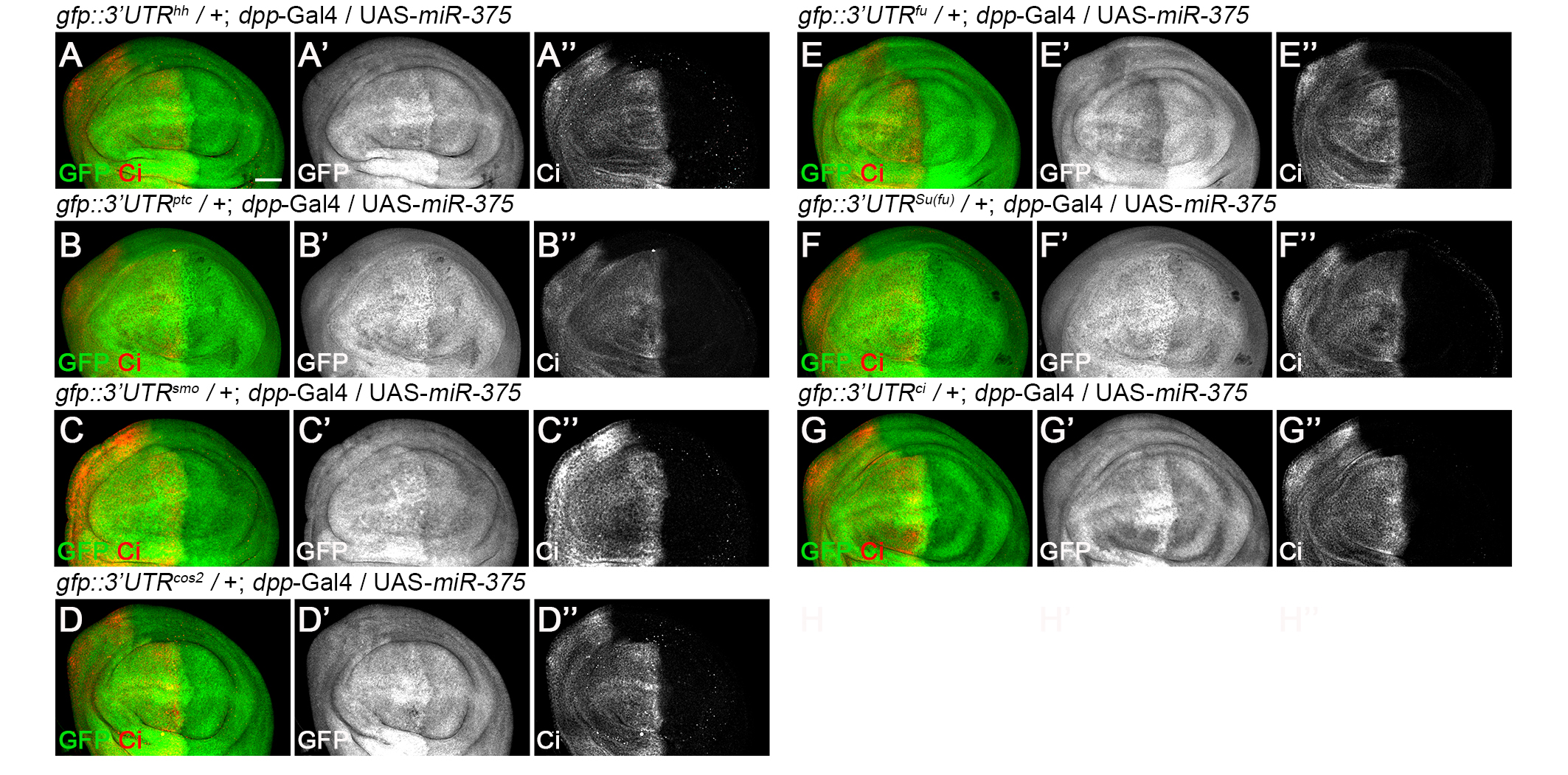

Supplement: Supplementary file 7 [file Image7.JPEG]

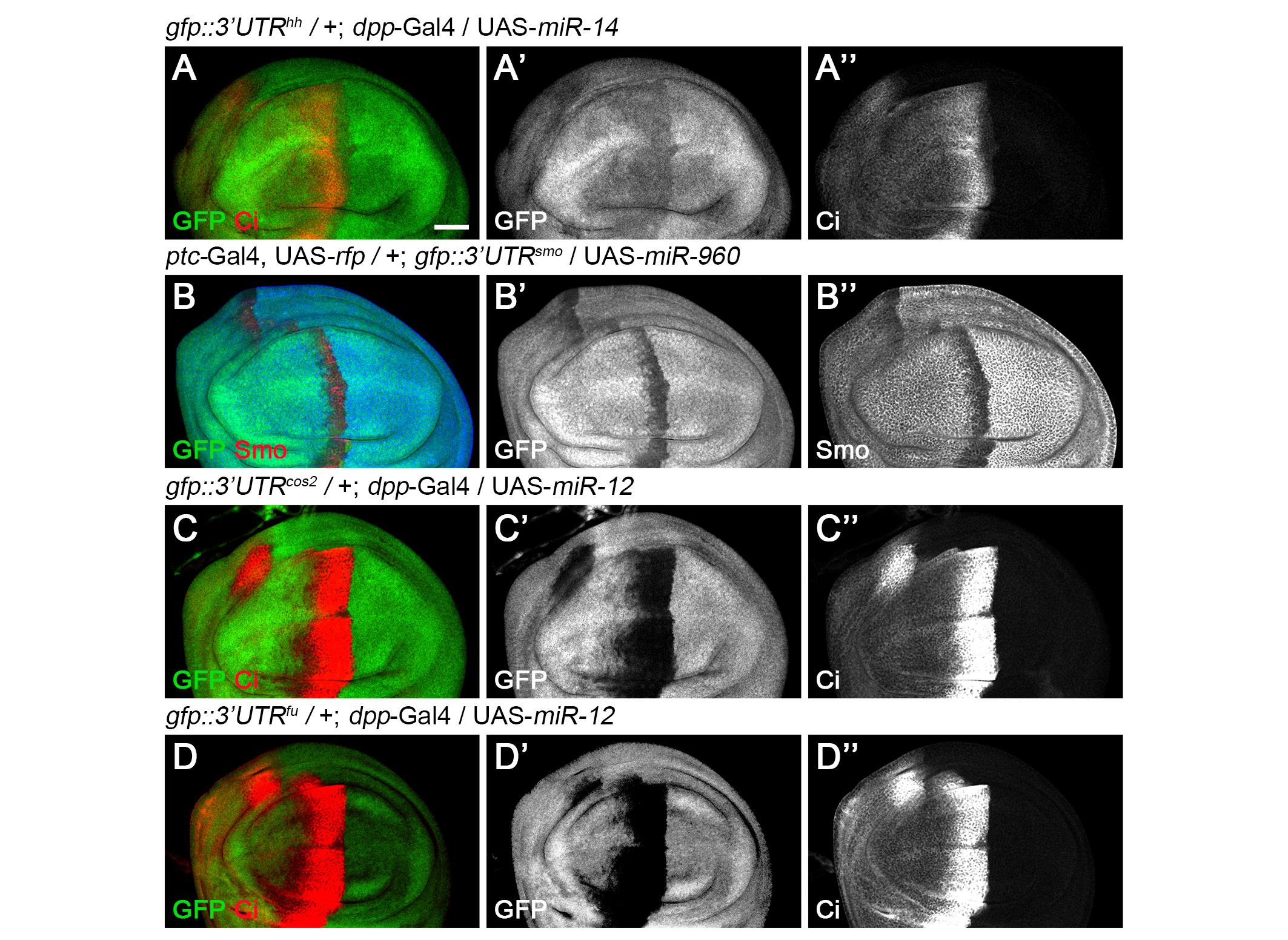

Supplement: Supplementary file 8 [file Image2.JPEG]

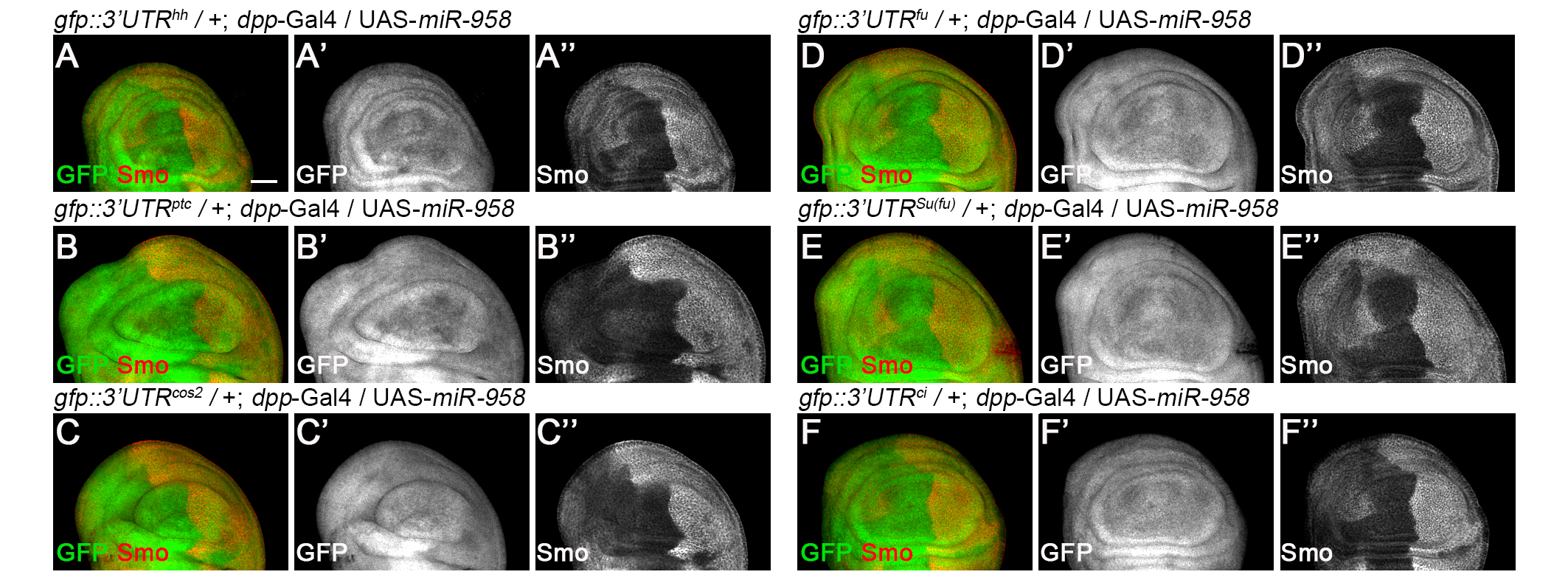

Supplement: Supplementary file 9 [file Image5.JPEG]

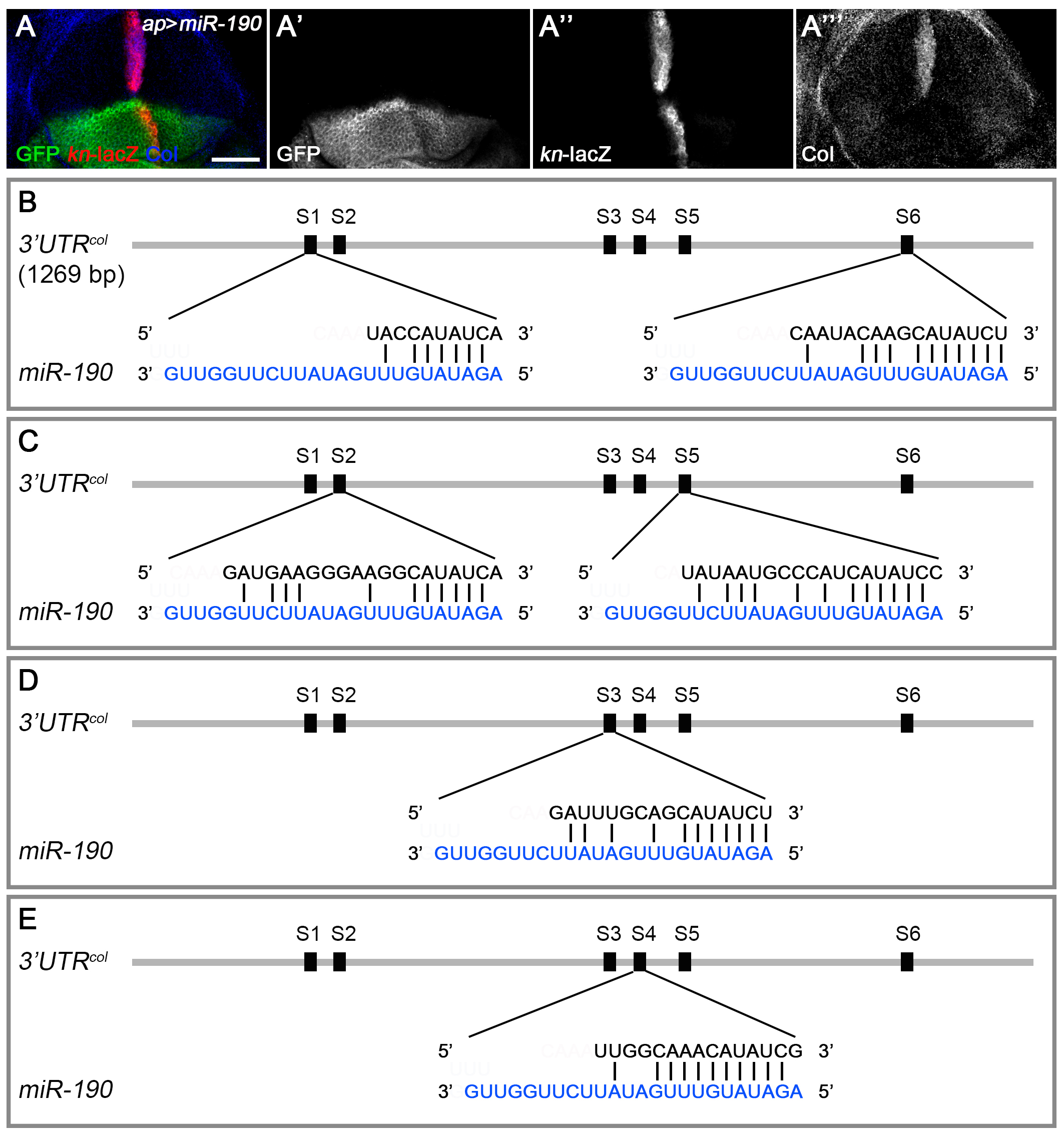

Supplement: Supplementary file 10 [file Image10.JPEG]

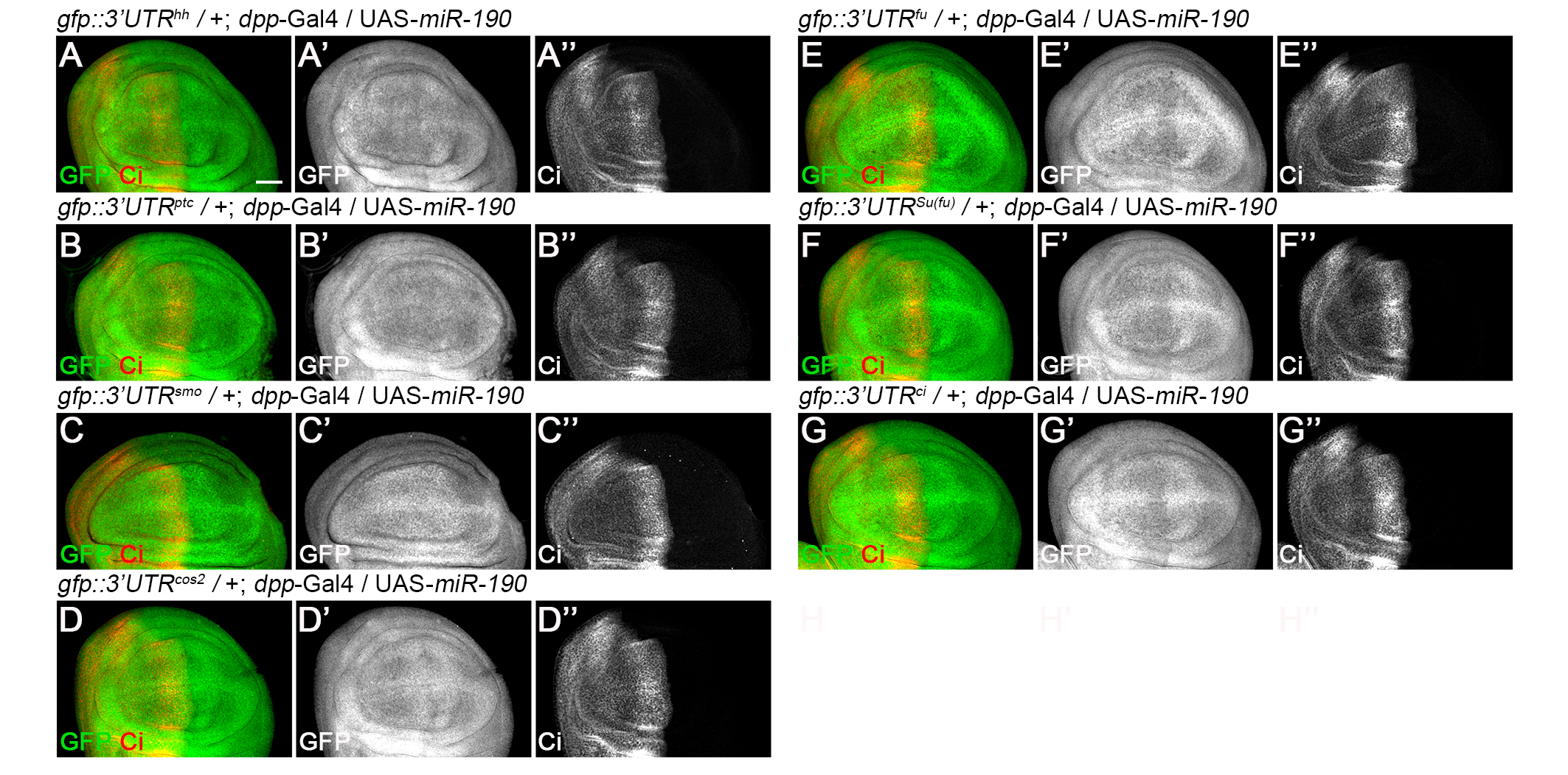

Supplement: Supplementary file 11 [file Image11.JPEG]

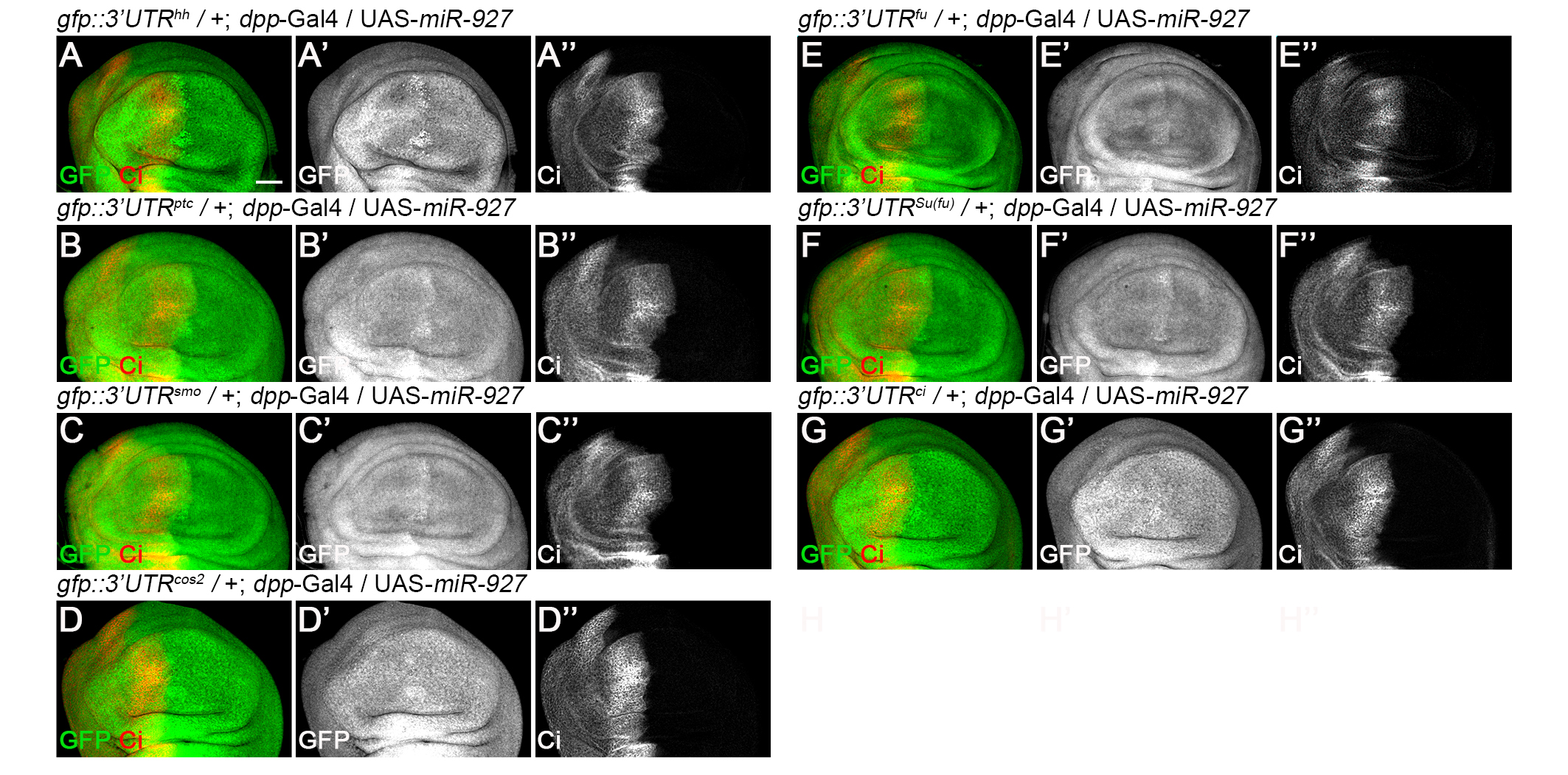

Supplement: Supplementary file 15 [file Image8.JPEG]

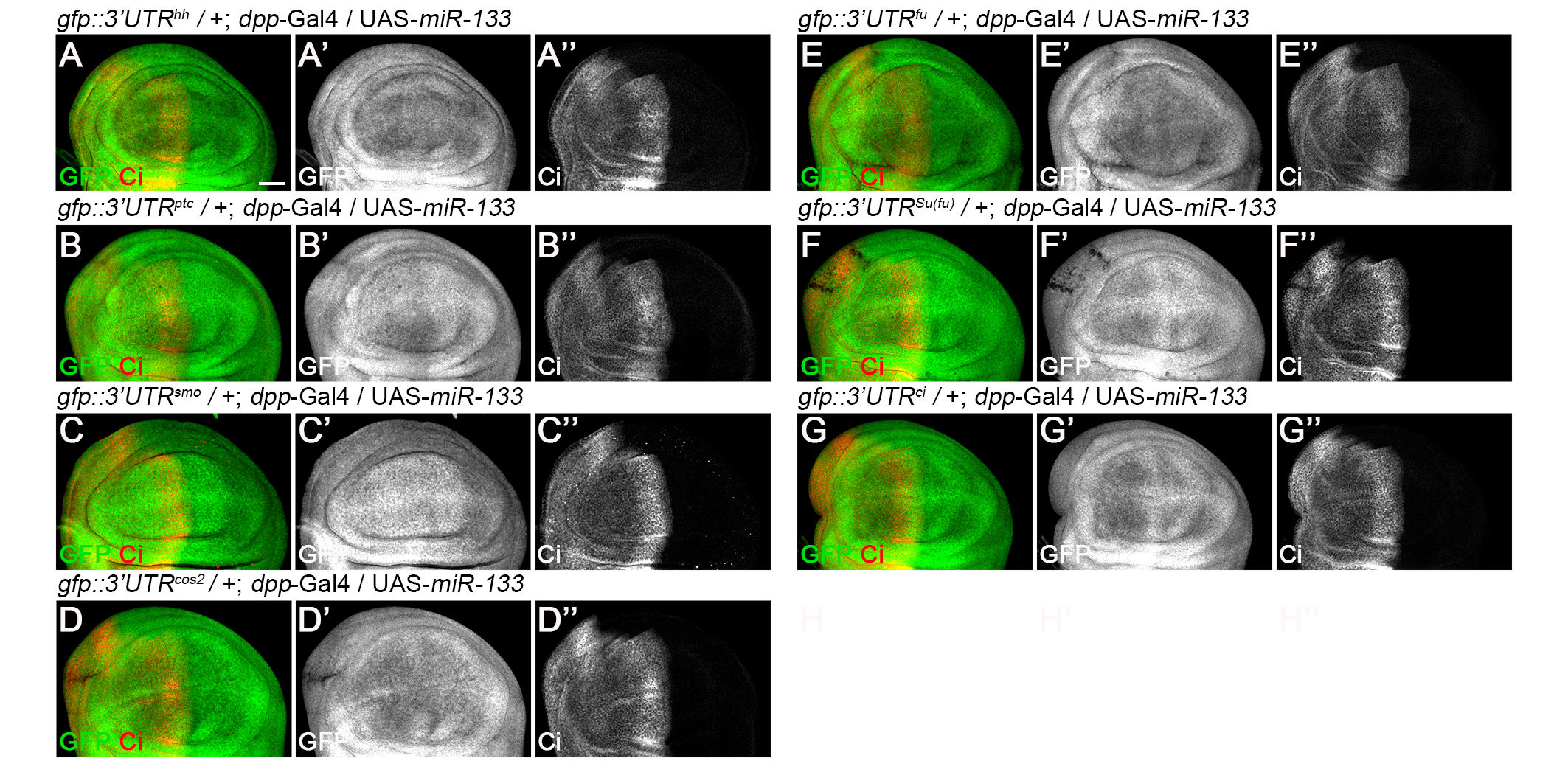

Supplement: Supplementary file 17 [file Image6.JPEG]
