# Supplementary material for: Dissection of the microRNA Network Regulating Hedgehog Signaling in Drosophila
Source: Front Cell Dev Biol. 2022 Apr 28;10:866491. doi: 10.3389/fcell.2022.866491 (PMC9096565; doi:10.3389/fcell.2022.866491)
Supplement: Supplementary file 14 [file Table4.DOCX]

**Supplementary Table 4. Genetic crosses for figures and supplemental figures.**

| Figures | Genetic crosses and conditions |
| --- | --- |
| Figure 3,E-E’’ | *ap*-Gal4, UAS-*mCD8-gfp* / +; UAS-*miR-932* / *kn*-lacZ at 18°C. |
| Figure 5,E-E’’ | *ap*-Gal4, UAS-*mCD8-gfp* / +; UAS-*miR-927* / *kn*-lacZ at 18°C. |
| Figure 5,F-F’’’ | *ap*-Gal4, UAS-*mCD8-gfp* / +; UAS-*miR-927* / *+* at 18°C. |
| Figure 6,A-A’’ | *ap*-Gal4, UAS-*mCD8-gfp* / +; UAS-*miR-958* / *kn*-lacZ at 18°C. |
| Figure 6,B-B’’’ | *ap*-Gal4, UAS-*mCD8-gfp* / +; UAS-*miR-958* / *+* at 18°C. |
| Figure 8,C-C’’ | *ap*-Gal4, UAS-*mCD8-gfp* / +; UAS-*miR-964* / *kn*-lacZ at 18°C. |
| Figure 8,D-D’’’ | *ap*-Gal4, UAS-*mCD8-gfp* / +; UAS-*miR-964* / *+* at 18°C. |
| Figure 9,A-A’’ | *hs*-*flp^122^* / +;; *Act5C*>*yw*>Gal4, UAS-*gfp* / UAS-*miR-10*. Larvae were heat shocked at 37°C for 10 minutes at 3 days after egg laying. |
| Figure 9,B-B’’ | *hs*-*flp^122^* / +;; *Act5C*>*yw*>Gal4, UAS-*gfp* / UAS-*miR-133*. Larvae were heat shocked at 37°C for 10 minutes at 4 days after egg laying. |
| Figure 9,C-C’’ | *hs*-*flp^122^* / +;; *Act5C*>*yw*>Gal4, UAS-*gfp* / UAS-*miR-190*. Larvae were heat shocked at 37°C for 10 minutes at 4 days after egg laying. |
| Figure 9,D-D’’ | *hs*-*flp^122^* / +;; *Act5C*>*yw*>Gal4, UAS-*gfp* / UAS-*miR-375*. Larvae were heat shocked at 37°C for 10 minutes at 4 days after egg laying. |
| Figure 9,E-E’’ | *hs*-*flp^122^* / +;; *Act5C*>*yw*>Gal4, UAS-*gfp* / UAS-*miR-927*. Larvae were heat shocked at 37°C for 10 minutes at 3 days after egg laying. |
| Figure 9,F-F’’ | *hs*-*flp^122^* / +;; *Act5C*>*yw*>Gal4, UAS-*gfp* / UAS-*miR-958*. Larvae were heat shocked at 37°C for 10 minutes at 4 days after egg laying. |
| Figure 9,G-G’’ | *hs*-*flp^122^* / +;; *Act5C*>*yw*>Gal4, UAS-*gfp* / UAS-*miR-964*. Larvae were heat shocked at 37°C for 10 minutes at 5 days after egg laying. |
| Supplementary Figure 3,A-A’’ | *ap*-Gal4, UAS-*mCD8-gfp* / +; UAS-*miR-5* / *+* at 29°C. |
| Supplementary Figure 3,B-B’’ | *ap*-Gal4, UAS-*mCD8-gfp* / *gfp::3’UTR^smo^*; UAS-*miR-5* / + at 29°C. |
